# Chlorpyrifos and Chlorpyrifos-Oxon: A Widening Spectrum of Toxicity

**DOI:** 10.3390/ijms27093909

**Published:** 2026-04-28

**Authors:** Sebastian Kalenik, Agnieszka Zaczek, Aleksandra Rodacka

**Affiliations:** 1 Department of Oncobiology and Epigenetics, Faculty of Biology and Environmental Protection, University of Lodz, 90-237 Lodz, Poland; sebastian.kalenik@edu.uni.lodz.pl (S.K.); agnieszka.zaczek@biol.uni.lodz.pl (A.Z.); 2 Doctoral School of Exact and Natural Sciences, University of Lodz, 90-237 Lodz, Poland

**Keywords:** chlorpyrifos, chlorpyrifos-oxon, pesticide, neurotoxicity, environmental toxicology, public health

## Abstract

Chlorpyrifos (CP) remains one of the most globally pervasive organophosphorus pesticides, and its toxicological profile continues to raise substantial public health and environmental concerns. While traditionally characterized by its potent acetylcholinesterase-inhibitory properties, accumulating evidence now shows that chlorpyrifos and its bioactive metabolite, chlorpyrifos-oxon (CPO), exert far broader toxic effects, including the induction of oxidative stress, enhancement of neuroinflammatory processes, and the triggering of persistent epigenetic alterations. In this review, we synthesize current findings to highlight the expanding spectrum of CP-induced toxicity, while also providing a multidisciplinary overview of chlorpyrifos characteristics, including its environmental fate, metabolism, and transformation pathways. The analysis encompasses not only classical neurotoxicity but also disruptions in neurodevelopment, endocrine signaling, gut microbiota composition, hepatic function, musculoskeletal integrity and carcinogenic pathways. By synthesizing results across human, animal, and environmental studies, this review offers a comprehensive overview of CP’s multidimensional toxicity and highlights the urgent need for improved biomonitoring, regulatory harmonization, and global strategies to reduce exposure.

## 1. Introduction

Organophosphorus compounds (OPs), originally synthesized in 1938 by German scientists and introduced as chemical warfare agents during World War II, were subsequently applied in agriculture after the war [1]. Due to their effectiveness in pest control, organophosphates replaced chlorinated insecticides and became today’s dominant plant protection agents. This broad class encompasses several chemical sub-groups, most notably phosphorothioates, phosphonates, and phosphates. Widely recognized examples include malathion, parathion, and diazinon, as well as compounds frequently scrutinized in the regulatory literature such as dichlorvos and chlorpyrifos-methyl [2]. Organophosphate insecticides account for nearly half of all used globally, with chlorpyrifos being the most widely utilized [3]. Introduced to the US market in 1965, chlorpyrifos (CP) became one of the most widely sold pesticides worldwide by the 1990s [4].

CP was extensively used in agricultural crops such as corn, soybeans, apples, and grapes, as well as in non-agricultural settings, including golf courses and residential areas [5]. However, numerous studies have demonstrated its neurotoxic, endocrine-disrupting, and developmental effects, leading to restrictions and bans on its use [6]. In the European Union, CP was withdrawn from the market in 2020 [7], and in the United States, its use was prohibited beginning in 2022 [8]. Despite these regulatory measures, residues of CP are still detected in food, water, and biological samples, as confirmed by European Food Safety Authority (EFSA) monitoring reports [9]. In this context, the 2025 decision of the Stockholm Convention Parties, increasing the number of exemptions for chlorpyrifos from seven to 22 recommended by POPRC, may further exacerbate public health risks [10].

A report published by EFSA in 2021, based on data from 2019, showed that CP was one of the pesticides most frequently exceeding the acute reference dose (ARfD) in food products. These exceedances were recorded in products such as apples, peaches, tomatoes, spinach, and lettuce. Additionally, CP residues were identified in animal products, primarily in kidneys, likely due to their presence in animal feed. The report also highlighted cases of CP being used for purposes other than its approved applications, such as in apple and spinach crops, as well as instances of its unacceptable use in organic farming [11]. Notably, the subsequent report published in 2023 confirmed that CP was predominantly detected in broccoli grown in the European Union and in randomly sampled products at concentrations exceeding legal limits. It was also found in buckwheat and other pseudocereals from Bolivia [12].

Statistical data show that pesticide exposure in general causes serious consequences for human health. According to the WHO report, it is estimated that about 3 million people worldwide suffer from pesticide poisoning annually. Among them, about 220,000 deaths are recorded, and approximately 750,000 develop chronic diseases after exposure to pesticides [13]. Pesticide self-poisoning accounts for 14–20% of global suicides (140,000 deaths each year) [14,15,16]. Among the implicated agents, organophosphate pesticides are of particular concern due to their widespread availability and extensive agricultural use. Chlorpyrifos has been implicated in both intentional poisonings, including suicides and homicides [17]. Epidemiological evidence further supports this association, Lee et al. (2007) identified a twofold higher risk of death from external causes, including suicide, among chlorpyrifos applicators at the highest exposure levels, despite no overall increase in mortality [18]. Toxicological identification in such cases remains challenging, and the number of dedicated clinical studies is limited. Nevertheless, available evidence confirms the clinical relevance of chlorpyrifos poisoning. In Taiwan, intentional ingestion has been associated with acute respiratory failure and excessive salivation, with a reported mortality rate of 17% [19]. A separate study reported a mortality rate of 15%, identifying hypotension, respiratory failure, coma, and corrected QT interval prolongation as significant independent predictors of death [20].

The toxicity of chlorpyrifos is primarily mediated by its active metabolite, chlorpyrifos-oxon, which is formed via cytochrome P450-mediated desulfuration of the parent compound. CPO acts as a potent inhibitor of acetylcholinesterase (AChE) activity in both the central and the peripheral nervous system. In addition to this primary cholinergic mechanism, alterations in paraoxonase 1 (PON1) activity are often detected in blood serum. PON1 is responsible for detoxifying chlorpyrifos-oxon, and its reduced activity may increase susceptibility to toxicity. Furthermore, PON1 modulation has been linked to disturbances in oxidative balance, suggesting an indirect role in redox dysregulation [21]. Moreover, studies have demonstrated that CP exposure can induce airway hyperreactivity (AHR), as observed in both male and female rats. This effect was particularly pronounced in female rats, who exhibited AHR at lower CP doses and earlier time points compared to males, indicating a higher susceptibility. The development of AHR was shown to affect both proximal and distal airways, as evidenced by increased airway resistance and tissue elastance in response to vagal stimulation, suggesting that chlorpyrifos may impact pulmonary function through mechanisms beyond classical cholinesterase inhibition [22]. The literature reports also indicate that the metabolite CPO inhibits butyrylcholinesterase and can react with proteins, specifically forming adducts with tyrosine residues in blood proteins such as albumin [23]. Epidemiological studies have shown a strong association between CP exposure and the risk of neurological disorders, such as developmental delays in children and neurodegenerative diseases later in life [24]. Even low doses of CP that do not inhibit AChE have been shown to be associated with neurodevelopmental disorders, including autism spectrum disorders (ASD). However, the mechanisms of action of CP and its metabolites are not yet fully understood [25,26].

The widespread use of pesticides poses a serious threat to both human health and the environment. They pollute soil, water and air, destroy biodiversity and cause numerous diseases in humans [27]. To effectively protect public health and ecosystems, it is essential to reduce pesticide use and implement advanced biomonitoring systems that enable continuous tracking of their impact on the environment and living organisms [28,29].

This paper presents a comprehensive review of the chemical properties, toxicokinetics, and multi-organ toxic effects of chlorpyrifos—one of the most widely used organophosphate insecticides of the 20th and 21st centuries. The main exposure routes, mechanisms of action, and metabolic pathways of the compound are discussed, with particular emphasis on its neurotoxicity and impact on neurobehavioral development. Current data on liver damage, musculoskeletal disorders, carcinogenic potential, and environmental persistence of chlorpyrifos are also presented. The review highlights discrepancies between the results of independent and industry-sponsored studies and discusses the implications for public health and regulatory policy. Overall, this paper attempts to synthesize the current state of knowledge and identify key directions for further research and action.

## 2. Methods of Review

A narrative review approach was selected for this study to provide a comprehensive and holistic synthesis of the multidimensional toxicity and the health effects of chlorpyrifos and its metabolites. This format was deemed most appropriate given the broad, interdisciplinary scope of the subject matter, which integrates evidence from molecular toxicology, environmental epidemiology, and regulatory science. Furthermore, the significant methodological heterogeneity among the included studies, ranging from mechanistic in vitro assays to complex longitudinal human cohorts, required a flexible framework to qualitatively integrate disparate data types into a cohesive conceptual model. To ensure a structured evaluation, a literature search was performed using electronic databases, including PubMed, Web of Science, and Scopus, covering studies published up to 2026. Search terms included combinations of “chlorpyrifos”, “chlorpyrifos-oxon”, “organophosphates”, “toxicity”, “neurotoxicity”, “oxidative stress”, “epidemiology”, and “human exposure”. Both experimental (in vitro and in vivo) and epidemiological studies were considered.

Studies were selected based on their relevance to the topic, with particular emphasis on peer-reviewed articles investigating mechanisms of toxicity, health outcomes, and exposure assessment. Regulatory reports and risk assessment documents were also included where appropriate.

## 3. Chemical Structure and Physicochemical Properties

**Figure 1:**
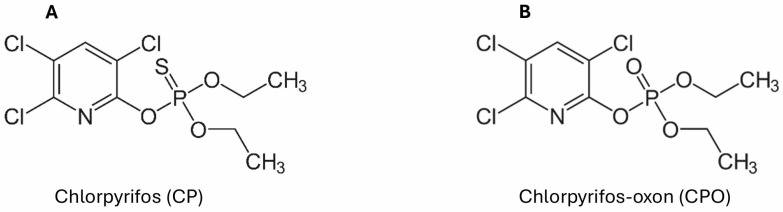
Chemical structure of (**A**) chlorpyrifos and (**B**) chlorpyrifos-oxon.

Chlorpyrifos (O,O-Diethyl O-(3,5,6-trichloropyridin-2-yl) phosphorothioate) is an insecticide belonging to the group of organophosphate pesticides. It appears as a colorless to white crystalline solid with a characteristic mild mercaptan (thiol) odor. While it is only slightly soluble in water, it dissolves readily in most organic solvents, such as acetone, xylene, and methanol [4,30]. The chemical formulas are shown in the figure (Figure 1).

The octanol–water partition coefficient (Kow) of chlorpyrifos is 4.7, suggesting a high capacity to cross cellular membranes as a result of its lipid-soluble nature, which is partly due to the presence of chlorine atoms in its structure. As a result, chlorpyrifos tends to bioaccumulate in living organisms [31].

Chlorpyrifos is also resistant to biodegradation in soil, with a half-life ranging from 60 to 120 days, depending on environmental conditions [32]. Its stability, as well as that of its metabolite chlorpyrifos-oxon, decreases with increasing pH. The compound remains stable in neutral and acidic aqueous solutions [33]. At pH 9, CP hydrolyzes with a half-life of about two weeks; at a more environmentally relevant pH of 7, hydrolysis is slower, with a half-life of 72–81 days. In soil, chlorpyrifos degrades slowly under both aerobic (half-life: 19–297 days) and anaerobic (half-life: 78–171 days) conditions. Similar findings were reported by Chai et al., who observed half-lives ranging from 157 to 257 days in soils with lower populations of microorganisms [34].

Due to its low volatility and slow aerobic degradation, chlorpyrifos tends to persist and spread widely in the environment, including soil, water and even the atmosphere [32] (Table 1). Notably, its environmental persistence can be exceptionally long, for instance, it has been detected in indoor air and soil as long as four and eight years after termiticide application [35,36,37].

## 4. Environmental Persistence

Chlorpyrifos can be transported through the atmosphere and deposited in various environmental compartments, including air, soil, water, and snow. Studies have confirmed its presence in air samples collected along the Mississippi River, in the United States, the Arctic, and remote regions of Antarctica and Greenland [39,40,41,42]. Chlorpyrifos is also among the most frequently detected insecticides in wet deposition samples collected from the Midwestern United States and the San Joaquin Valley, California. In the San Joaquin Valley, precipitation samples analyzed during storm events in January and February 2001 showed that the average concentration of chlorpyrifos was approximately 2.5 times higher than in stormwater runof–f, indicating atmospheric deposition as a significant source of contamination [43,44].

**Figure 2:**
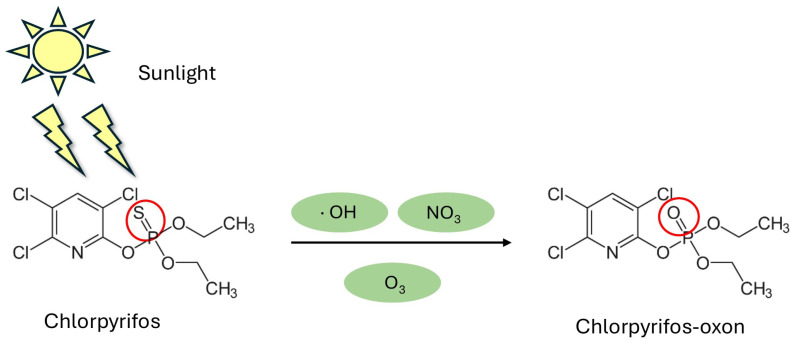
Atmospheric transformation pathways of chlorpyrifos. The diagram illustrates the abiotic oxidative desulfuration of chlorpyrifos into its primary metabolite—chlorpyrifos-oxon (CPO). This photochemical process is driven by solar radiation (Sunlight) and mediated by key atmospheric oxidants, including hydroxyl radicals (·OH), nitrate radicals (NO_3_), and ozone (O_3_). The transition from the parent compound to the oxon form is highlighted by the substitution of the sulfur atom (S) with an oxygen atom (O) within the phosphorothioate group.

In the atmosphere, CP undergoes photochemical reactions such as oxidation by hydroxyl radicals (·OH) and reactions with ozone and nitrate radicals (NO_3_), which contribute to its degradation and the formation of chlorpyrifos-oxon (CPO) (Figure 2). The atmospheric half-life of CP is approximately two hours (specially mediated by ·OH radicals), indicating that its presence and transport depend on environmental factors such as wind speed and direction, temperature, and UV radiation intensity [38,45]. In contrast, CPO exhibits a significantly longer atmospheric half-life, estimated at approximately 11 h. This increased persistence is linked to the lower reactivity of CPO towards hydroxyl radicals compared to the parent compound. However, it is important to note that CPO possesses a lower vapor pressure and higher water solubility than CP. Higher water solubility promotes its removal from the atmosphere through wet deposition and scavenging, thereby reducing its actual residence time. However, the relative chemical stability of CPO still allows for a prolonged presence in the air under dry conditions. Consequently, this extends the window for inhalation exposure and enhances its cumulative toxic potential for non-target organisms [38].

Due to its ability to undergo atmospheric transport, accumulate in soil, and deposit in aquatic ecosystems, chlorpyrifos poses a significant threat to both environmental and human health. As mentioned earlier, the relatively high log Kow value of CP (4.7) reflects its pronounced lipophilicity, which facilitates rapid penetration through biological membranes and accumulation in fatty tissues. Furthermore, its high octanol-air partition coefficient (log Koa = 8.3) indicates a strong affinity for organic phases in the atmosphere. This property promotes the adsorption of CP onto airborne particulates, enhancing its potential for atmospheric transport and increasing the risk of inhalation exposure in humans and wildlife. Although the relatively high log Kow suggests a strong bioaccumulation potential, experimentally determined whole-body bioconcentration factor (BCF) values for 17 species of freshwater and saltwater fish are more moderate. These values typically range from 396 to 5100, with a mean of 1129 and a geometric mean of 848, and in most cases remaining below 2000, indicating limited bioaccumulation potential. However, in *Danio rerio* eleutheroembryos, it reaches much higher levels, ranging from 3548 to 6918. This disparity is attributed to the underdeveloped metabolic capacity of embryos, which prevents the rapid biotransformation and excretion observed in adult organisms [38]. These findings suggest that while CP has the thermodynamic potential to partition into lipids, its actual accumulation in the food web is limited by rapid metabolic biotransformation and excretion, preventing the extreme biomagnification seen in more persistent organic pollutants [38,45,46].

The detection of chlorpyrifos in Arctic fauna indicates its ability to accumulate and increase in concentration along the food chain or environmental conditions [47]. Observations of detectable amounts of CP at distances exceeding 1000 km suggest that, under specific conditions, its environmental half-life is significantly extended. At higher latitudes, factors such as lower temperatures, limited photolysis, and smaller concentrations of hydroxyl radicals slow down the transformation of CP. This leads to a longer characteristic travel distance (CTD), estimated between 300 and 1000 km, as seen in significant concentrations measured in the Svalbard ice-cap [38]. Consequently, while CP is rapidly metabolized in temperate climates, its increased environmental persistence in the cold, rather than a failure of metabolic biotransformation, may account for its presence in Arctic biota [38].

Lavin et al. (2012) [48] reported CP in airborne particulate matter in the remote Southern Alps region of New Zealand, likely originating from the Canterbury Plains. While the compound can be transported in the gas phase over shorter distances, its association with atmospheric particulate matter (aerosols) significantly extends its transport potential. This is due to aerosol-mediated transport, which protects CP from rapid atmospheric degradation by shielding the molecules from photo-oxidation, allowing it to reach much greater distances where it degrades more slowly. Along with pesticides such as dieldrin and trans-chlordane, CP was classified as a persistent organic pollutant (POP) with the potential for long-range atmospheric transport, posing risks to the Antarctic environment [48]. Furthermore, it should be emphasized that the existing literature suggests the dissipation of CP is primarily driven by volatilization, accounting for approximately 53% to 70% of its loss. This high rate of evaporation underscores the role of foliar application as a significant source of CP entry into the atmospheric compartment, thereby facilitating widespread airborne exposure [49]. Chlorpyrifos has been detected in both indoor and outdoor environments. While its presence in household dust indicates the potential for direct human exposure, its accumulation in soil, water, and plants intensifies its harmful effects on terrestrial and aquatic organisms [50].

Given the toxicological and ecological concerns associated with CP and its metabolites, advanced detection methods are crucial for effective environmental monitoring. A promising technique involves the use of silver phosphate (Ag_3_PO_4_) nanozymes, which act as oxidative catalysts for selective CP detection. Unlike conventional horseradish peroxidase (HRP)-based methods, Ag_3_PO_4_ nanozymes offer greater stability and efficiency in detecting CP in various environmental matrices, making them a valuable tool for future monitoring efforts at the molecular level [51].

## 5. Pathways of Exposure and Metabolic Transformation

The toxicokinetics and health effects of chlorpyrifos are determined by the route, duration, and absorbed dose of exposure. Chlorpyrifos enters the human body through three primary routes: inhalation, dermal contact, and oral intake.

- Inhalation results in relatively rapid systemic absorption through the respiratory epithelium and is particularly relevant in occupational settings, including agricultural and greenhouse workers at pesticide application sites, where higher airborne concentrations may trigger acute cholinergic symptoms [52].
- Dermal exposure occurs mainly during the preparation and application of spray formulations or through contact with contaminated surfaces, soil, and plants. Although percutaneous absorption is generally slower, prolonged skin contact with concentrated products, especially without appropriate protective equipment, can lead to clinically significant internal doses.
- Oral exposure, associated with the consumption of contaminated food or water, represents the predominant pathway in the general population and is typically characterized by lower concentrations but potentially chronic low-dose intake through dietary residues [31].

Experimental studies confirm that the exposure route influences absorption kinetics and tissue distribution, with inhalation and oral intake usually producing faster systemic availability than dermal exposure [53]. Animal models further show that developing organisms are more susceptible than adults, exhibiting neurodevelopmental and synaptic alterations even at comparatively lower concentrations following subchronic or developmental exposure [54].

Both human and animal data indicate a clear dose–response relationship. Acute high-dose exposure, most often linked to inhalation or accidental ingestion, leads to marked acetylcholinesterase inhibition and overt neurotoxic symptoms [31]. In contrast, chronic low-level exposure, typically related to dietary or environmental sources, may not produce immediate poisoning signs but has been associated with subtle neurobehavioral, developmental, and endocrine effects [8,55]. Epidemiological studies additionally suggest that prenatal and early-life exposure, even at relatively low environmental levels, may be linked to impaired neurodevelopment and cognitive function [56]. Therefore, both exposure route and dose should be considered key modifiers of toxic response in risk evaluation.

Children constitute a particularly vulnerable population due to combined behavioral and physiological factors. Frequent hand-to-mouth activity and relatively higher dietary intake of fruits and vegetables, as well as immature intestinal and enzymatic systems contribute to increased exposure and reduced detoxification capacity [52,57,58,59]. Table 2 provides a comparative overview of real-world chlorpyrifos concentrations and estimated daily doses across different environmental and occupational scenarios.

**Figure 3:**
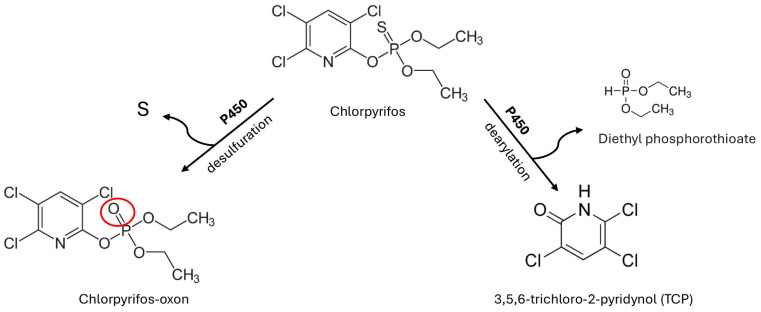
Biotransformation pathways of chlorpyrifos by cytochrome P450. The diagram illustrates the two competing metabolic routes for CP in the liver. One pathway involves the oxidative desulfuration of the parent compound, leading to the formation of the potent acetylcholinesterase inhibitor, chlorpyrifos-oxon. In this activation step, the sulfur atom is replaced by an oxygen atom. Parallel to this, a detoxification pathway occurs through oxidative dearylation. This reaction involves the cleavage of the phosphorothioate ester bond, yielding the non-toxic metabolites diethyl phosphorothioate and 3,5,6-trichloro-2-pyridinol (TCP). Both competing pathways are catalyzed by specific isoforms of the cytochrome P450 (P450) enzyme system.

Upon entering the organism, CP is primarily metabolized in the liver, a process catalyzed by cytochrome P450 enzymes, particularly the CYP2B6, CYP2C19, and CYP3A4 isoforms [66,67,68]. The biotransformation of chlorpyrifos occurs in several stages:

(1) Oxidation—The initial step involves oxidative desulfuration, catalyzed by cytochrome P450, leading to the formation of chlorpyrifos-oxon, a more potent inhibitor of acetylcholinesterase. Cytochrome P450 also contributes to CP detoxification through oxidative dearylation, producing 3,5,6-trichloro-2-pyridinol (TCP) (Figure 3) [69,70,71].
(2) Hydrolysis—Both CP and CPO undergo hydrolysis, catalyzed by phosphotriesterases (specifically A-esterases) and arylesterases, including paraoxonase 1 (PON1). It is important to note that PON1 acts as a phosphotriesterase in this context, rather than a phosphodiesterase. This reaction yields 3,5,6-trichloro-2-pyridinol (TCP) and diethyl thiophosphate (DETP) [72,73]. In the biochemical framework of OP metabolism, A-esterases like PON1 are responsible for the hydrolysis of OPs, whereas B-esterases, such as AChE and BChE, are the targets of their inhibition. The efficiency of this catalytic hydrolysis depends on genetic polymorphisms of PON1 (e.g., Q192R), which influence an individual’s capacity for detoxifying OPs such as CP.
(3) Conjugation—The primary metabolites, TCP and DETP, undergo phase II metabolism, during which they are conjugated with glucuronic acid or sulfate. These modifications increase water solubility, facilitating renal elimination [30].

Due to its lipophilic nature, chlorpyrifos is widely distributed in the organism, with a tendency to accumulate in adipose tissue. However, its systemic persistence is limited by rapid hepatic metabolism, resulting in low and transient concentrations of the parent compound in blood. In contrast, its metabolites, particularly TCP and DETP, are more stable and dominate in systemic circulation. These compounds are subsequently distributed to organs such as the liver, kidneys, and brain, but do not significantly bioaccumulate due to efficient metabolic clearance [74].

Studies reported that in rats, the biological half-life of CP is up to 24 h in the blood and up to 60 h in adipose tissue [75]. Experimental data indicate that CP is rapidly distributed to highly perfused organs, including the liver, brain, and kidneys [74]. While its lipophilic nature leads to preferential accumulation in adipose tissue [76,77], its metabolites have also been detected in the liver, brain, kidneys and ovaries [53,78]. However, the parent insecticide is difficult to quantify in blood due to its rapid metabolism to 3,5,6-trichloro-2-pyridinol (TCP). Pharmacokinetic studies in rats demonstrate that major differences in the kinetics of its metabolites (TCP and DETP) occur primarily within the first 3 h post-exposure, reflecting the time required for hepatic CYP450-mediated metabolism. After this initial phase (≥3 h), the blood concentrations of CP-derived metabolites stabilize and become comparable to those observed following direct exposure to the metabolites themselves [74].

In rats, TCP is readily eliminated, primarily in a conjugated form (glucuronide or sulfate), which accounts for 70–83% of the urinary recovery, while free TCP accounts for approximately 21%. The terminal phase half-life for TCP derived from CP is approximately 6.3 h in rats, slightly longer than for directly administered TCP (3.8 h) due to the ongoing metabolic formation from the parent compound. Regarding dialkylphosphates, DETP is formed via dearylation; while excreted in urine, its recovery is lower (approximately 22%), and it exhibits a longer terminal half-life of 34–38 h. The efficient renal clearance of these polar conjugates is the final step in the detoxification process, preventing the systemic accumulation of reactive intermediates. While TCP is rapidly eliminated, the significantly longer half-life of DETP suggests a slower fractional excretion, which may be attributed to differences in renal tubular reabsorption or protein binding. This underscores that the urinary excretion profile is not only a result of hepatic metabolism but also of the specific renal handling of each metabolite [74].

(4) Excretion—Chlorpyrifos metabolites are excreted primarily via urine, with species-specific differences in the elimination profile:
  - Humans: Approximately 70% of an absorbed dose of CP is excreted as TCP in urine within five days after acute oral ingestion [79]. The pharmacokinetic half-life of 3,5,6-TCP in urine is about 27 h following oral exposure and 18 h after dermal exposure. Dermal absorption of CP is relatively low, ranging from 1% to 3%.
  - Rats: CP is efficiently metabolized, with approximately 84% of the administered dose excreted via urine and 5% via feces within 72 h. No unchanged parent compound is detected in urine. The dominant urinary metabolites are 3,5,6-TCP and its glucuronic and sulfuric acid conjugates [30].

**Figure 4:**
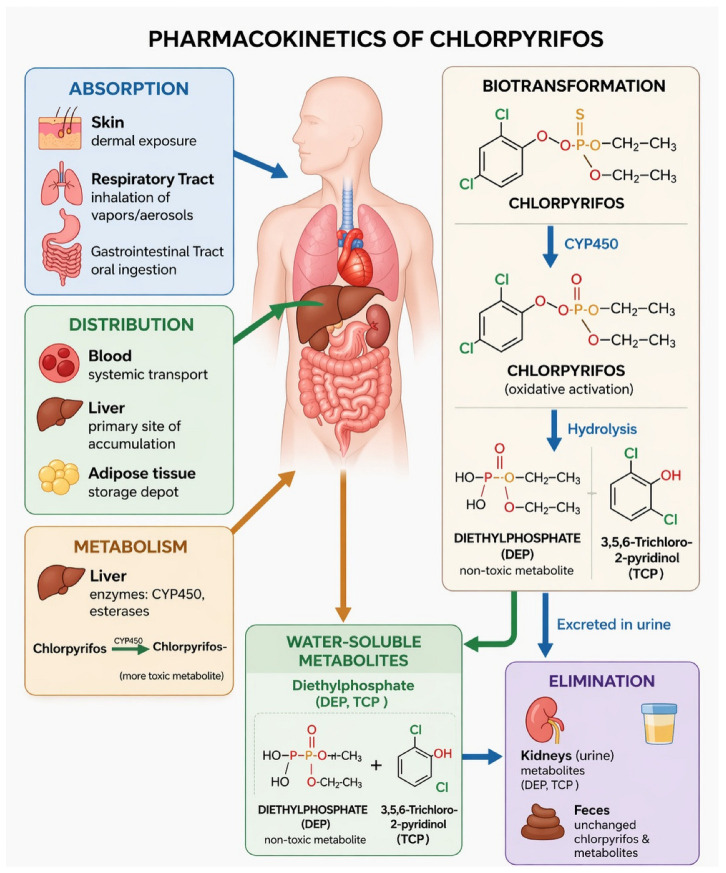
Pharmacokinetics of chlorpyrifos in humans: the compound is absorbed via the skin, respiratory tract, and gastrointestinal tract, and is subsequently distributed through the bloodstream primarily to the liver and adipose tissue. In the liver, it undergoes biotransformation by CYP450 enzymes to a more reactive form, followed by hydrolysis to water-soluble metabolites (DEP and TCP). These metabolites are mainly excreted in urine, while small amounts of the parent compound and its metabolites may be eliminated in feces.

Beyond metabolic pathways and elimination profiles, substantial interspecies, age-, and dose-dependent differences in sensitivity exist, largely due to species-specific toxicokinetic processes, including variations in absorption, distribution, metabolism, and excretion (ADME) [78,80]. For example, dermal absorption in humans is minimal (~1–3% of an applied dose), whereas oral absorption can reach ~70%, highlighting the importance of exposure route in determining systemic burden. Notably, humans generally exhibit lower PON1 activity than rodents, resulting in reduced detoxification of the highly toxic metabolite chlorpyrifos-oxon and, increasing susceptibility to neurological effects [74]. Age is another critical determinant of vulnerability, as age-dependent variability in enzymes such as PON1 and CYP450 enhances sensitivity to organophosphate exposure in infants and children [81,82,83,84]. Although data on sex differences in humans are limited, studies in non-human mammals indicate that baseline cholinesterase activities and pharmacokinetic profiles can differ between males and females, potentially affecting CP metabolism and biological effects (Figure 4) [85,86].

Collectively, these interspecies and developmental differences underscore the importance of carefully considering toxicokinetic variability when extrapolating chlorpyrifos toxicity data from animal models to human health risk assessment [21]. Additionally, the possibility of environmental transformation of CP into the toxic compound CPO as a result of its reaction with hypochlorous acid formed during drinking water disinfection by chlorination was described. This transformation is a critical factor in human exposure through treated water systems [87].

## 6. Molecular Mechanisms of Toxicity

The primary toxic mechanism of chlorpyrifos involves the inhibition of acetylcholinesterase activity. This enzyme catalyzes the hydrolysis of acetylcholine (ACh) into choline and acetate, a process essential for proper nerve signal transmission at synapses [88]. CP binds to the serine residue in the active site of AChE, phosphorylating it and thereby blocking the enzyme’s activity. As a result, ACh accumulates within cholinergic synapses, causing excessive stimulation of both nicotinic and muscarinic receptors. In insects, where ACh serves as the primary excitatory neurotransmitter, the excess of acetylcholine leads to overstimulation of receptors, disruption of neuromuscular transmission, paralysis, and ultimately death [89,90,91].

**Figure 5:**
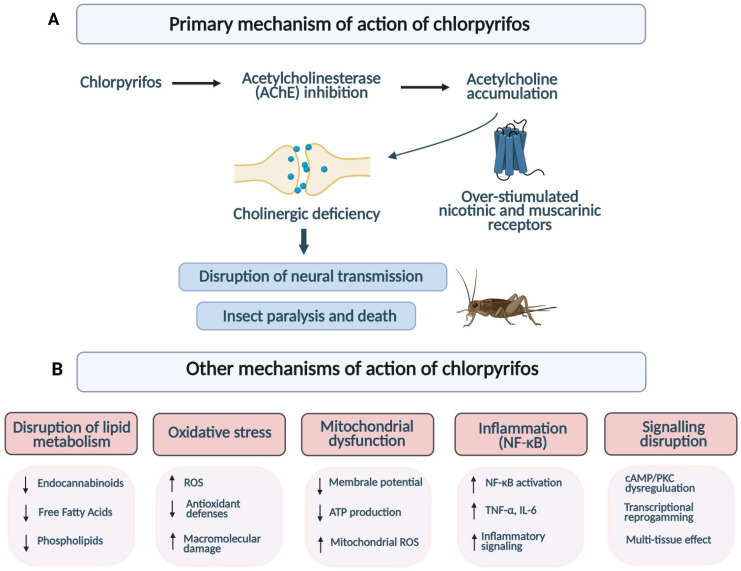
Schematic representation of the primary and other mechanisms of action underlying chlorpyrifos-induced neurotoxicity. The diagram summarizes the dual pathways of CP-mediated toxic effects. (**A**) The primary mechanism (top section) involves the irreversible inhibition of acetylcholinesterase, leading to the pathological accumulation of acetylcholine at the synapse. This results in the overstimulation of nicotinic and muscarinic receptors, causing disruption of neural transmission and leading to paralysis and death, as exemplified in the insect model. (**B**) The other mechanisms (bottom section) highlight non-cholinergic pathways of toxicity, including, oxidative stress, mitochondrial dysfunction, inflammation (NF-κB activation), disruption of intracellular signaling pathways, and alterations in lipid metabolism. These processes are associated with increased reactive oxygen species production, impaired energy metabolism, activation of pro-inflammatory mediators and dysregulation of key signaling cascades.

However, the toxicity of CP is not limited to the classical inhibition of AChE. Studies indicate additional molecular targets and mechanisms of action. It has been demonstrated that CP can disrupt brain lipid metabolism by inhibiting the activity of serine hydrolases such as monoacylglycerol lipase (MAGL) and fatty acid amide hydrolase (FAAH) [92]. These enzymes regulate endocannabinoid signaling and lipid homeostasis, and their inactivation disrupts neuronal function. Furthermore, CP exposure has been associated with a reduction in specific free fatty acids (FFA) and phospholipid metabolites in neural tissues, highlighting the involvement of lipid signaling pathways in its neurotoxic effects [92,93]. These primary and other mechanisms are schematically summarized in Figure 5.

A growing body of evidence indicates that CP induces oxidative stress through excessive generation of reactive oxygen species and impairment of antioxidant defense systems, including reduced expression and activity of key enzymes such as catalase, superoxide dismutase, and glutathione peroxidase, ultimately leading to cellular damage and genotoxic effects [7,94,95,96,97,98]. Furthermore, oxidative stress induced by CP has been associated with activation of the Nrf2 signaling pathway and increased expression of Nrf2-dependent proteins, including heme oxygenase-1 (HO-1) [95,99,100,101].

Oxidative stress induced by CP is closely linked to mitochondrial dysfunction. CP exposure has been shown to disrupt mitochondrial membrane potential, impair ATP production, and increase mitochondrial ROS generation, ultimately triggering apoptotic pathways. This interplay between oxidative stress and mitochondrial impairment may contribute to a self-perpetuating cycle of cellular damage [94,100,102,103].

In addition to its pro-oxidant effects, CP activates inflammatory responses through the NF-κB signaling pathway, a key transcriptional regulator of pro-inflammatory cytokine expression. CP-induced NF-κB activation leads to increased production of TNF-α and IL-6, as demonstrated both in in vitro microglial models and in in vivo mammalian tissues, indicating that the inflammatory response is not restricted to a single cell type [104,105]. Elevated levels of these cytokines may further amplify tissue damage by potentiating oxidative stress and sustaining inflammatory signaling, thereby contributing to the chronic toxic effects associated with CP exposure.

CP also disrupts intracellular signaling pathways essential for cellular homeostasis, most notably the adenylyl cyclase (AC)/cAMP cascade and protein kinase C (PKC) signaling. Exposure to CP elicits broad transcriptional reprogramming of genes involved in cAMP-mediated signaling, apoptosis, and oxidative stress response [106,107]. Concurrently, PKCδ, a redox-sensitive isoform highly expressed in neuronal cells, undergoes proteolytic activation upon CP exposure, generating a constitutively active catalytic fragment that promotes apoptotic cell death through caspase-3-dependent mechanisms [108]. The resulting dysregulation of these cascades impairs gene expression programs, compromises synaptic integrity, and undermines cell survival.

Notably, CP-induced perturbations of intracellular signaling are not restricted to the nervous system; convergent evidence from cardiac and hepatic models demonstrates that disruption of cAMP and PKC pathways represents a broadly conserved mechanism of CP cytotoxicity across multiple tissue types [109].

The convergence of these molecular perturbations, oxidative stress, mitochondrial dysfunction, persistent inflammation, and impaired intracellular signaling, creates conditions that inherently promote genomic instability. This is well supported by the available experimental evidence, as discussed below.

In summary, the action of chlorpyrifos involves not only the classical inhibition of AChE but also disturbances in lipid homeostasis and alternative cellular signaling mechanisms that may potentiate its neurotoxic effects.

### Genotoxic Effects of Chlorpyrifos

Based on the mechanisms described above, in particular the ability of CP to induce oxidative stress and mitochondrial dysfunction, chlorpyrifos has been shown to exert significant genotoxic effects in a wide range of in vitro and in vivo models. These effects encompass multiple levels of genomic damage, including primary DNA damage, chromosomal instability, and epigenetic changes.

Numerous studies have demonstrated that chlorpyrifos induces DNA strand breaks in a concentration- and time-dependent manner. The alkaline comet assay revealed significant increases in single-strand DNA breaks in multiple cell types and tissues following exposure to sublethal concentrations [110,111,112]. Consistent with these findings, in vivo studies have shown dose-dependent increases in DNA damage in liver, blood, and gill cells of exposed organisms [111,112,113]. Evidence also indicates the capacity of CP to induce double-strand breaks [114,115].

Chromosomal instability constitutes an additional key aspect of CP-induced geno-toxicity. In vivo micronucleus assays in Wistar rats demonstrated that oral exposure to sublethal doses of CP induced significant increases in micronucleated polychromatic erythrocytes in bone marrow [116,117]. Similarly, exposure of freshwater fish to sublethal CP concentrations resulted in significant, time- and concentration-dependent increases in micronuclei frequency in erythrocytes and DNA strand breaks in lymphocytes and gill cells [111]. Khan et al. (2022) assessed the genotoxic and cytotoxic potential of CP at permissible regulatory concentrations in juvenile common carp (*Cyprinus carpio*), demonstrating that even sub-lethal exposure induces significant behavioral impairments and micronuclei formation in a dose- and time-dependent manner [118]. Furthermore, in a separate study, low-dose CP exposure did not increase micronuclei frequency but significantly elevated, in a dose- and time-dependent manner, the occurrence of other nuclear abnormalities, including notched, blebbed, lobed, and eight-shaped nuclei, as well as nuclear buds, bridges, and binucleated cells [119].

Želježić et al. (2016) [120] assessed the cytotoxic and genotoxic effects of CP in human peripheral blood lymphocytes and HepG2 cells at toxicologically relevant, real-life exposure concentrations. CP demonstrated significant DNA-damaging potential. Oxidative stress biomarkers were not significantly altered, suggesting that the genotoxic effects were mediated by direct interactions between CP and/or its metabolites and the DNA structure [120]. Sex-dependent differences in genotoxic susceptibility have also been reported, with male rats showing significantly greater frequencies of binucleated cells and DNA strand breaks in the comet assay than females following equivalent CP exposure [121].

Beyond direct DNA damage and chromosomal instability, CP has also been shown to alter gene expression through epigenetic mechanisms, without directly modifying the DNA sequence itself. Accumulating evidence indicates that CP affects multiple levels of epigenomic regulation, including DNA methylation, histone modifications, and non-coding RNA expression, with consequences ranging from hepatic carcinogenesis to metabolic dysfunction and neurodevelopmental disorders [122,123,124,125].

At the level of DNA methylation, in vitro studies on human normal liver cells (WRL-68) demonstrated that chronic CP exposure drives epigenetic reprogramming through dose-dependent downregulation of DNA methyltransferases DNMT-3A and DNMT-3B, leading to genome-wide disruption of DNA methylation. This dysregulation resulted in aberrant expression of genes governing cell cycle progression, DNA repair (*BRCA1*, *BRCA2*, *TP53*, *ATM*), epithelial-to-mesenchymal transition (EMT), and apoptosis, collectively facilitating neoplastic transformation and underscoring CP’s potential as a liver carcinogen [125]. Epidemiological evidence further supports the relevance of CP-induced DNA methylation changes in human populations. A birth cohort study demonstrated that prenatal CP exposure was significantly associated with increased methylation of the PPARγ gene promoter in placental tissue, and that higher methylation levels correlated with poorer cognitive and language development in children at two years of age, particularly in boys [126]. Consistent with these findings, in vivo exposure of mice to CP downregulated key regulators of DNA methylation in hepatic tissue, including *Dnmt1*, *Mthfr*, and *Tet2*, further demonstrating that CP disrupts epigenetic homeostasis across multiple experimental models [127].

CP-induced epigenetic dysregulation extends beyond the liver and encompasses non-coding RNA-mediated mechanisms, particularly in the central nervous system. Changes in miRNA expression across brain regions play critical roles in neuronal differentiation, development, survival, and function, regulating neurobehavioral processes such as learning, memory, and synaptic plasticity, while also contributing to neuroprotection against neurodegenerative diseases [128,129]. Dysregulation of the miR-132/212 cluster has been linked to impaired neuronal development and the onset of degenerative conditions including Alzheimer’s disease, Parkinson’s disease, epilepsy, Rett syndrome, and psychiatric disorders such as schizophrenia [129,130]. In rats exposed to CP, upregulation of miR-132 and miR-212 in the hippocampus suggests their involvement in neurotrophin-dependent cognitive dysfunction and synaptic deficits, contributing to the pathogenesis of multiple neurological disorders [131,132,133]. Members of the miR-181 family are particularly relevant to CP-induced neuroinflammatory and oxidative pathways [134] and have been implicated in neurodegenerative and inflammatory CNS disorders including multiple system atrophy [135,136,137]. Elevated miR-19a levels have additionally been observed in ALS models, while miR-181a dysregulation has been linked to amyloid pathology and cognitive decline in Alzheimer’s disease [138].

The mechanistic basis of miR-181-mediated neurotoxicity has been characterized in human SH-SY5Y neuroblastoma cells, where CP activates the miR-181/SIRT1/PGC-1α/Nrf2 pathway. CP-induced upregulation of miR-181 suppresses SIRT1 expression, a key regulator of cellular stress response, leading to reduced PGC-1α and Nrf2 levels, impaired antioxidant defense, and excessive ROS accumulation. This pathway dysregulation further triggers activation of pyroptosis-associated proteins, NLRP3, caspase-1, IL-1β, and IL-18, resulting in inflammatory cell death [139].

Collectively, the studies discussed above indicate that chlorpyrifos is a multifaceted genotoxic agent whose harmful effects extend far beyond acetylcholinesterase inhibition to include direct DNA strand breaks, chromosomal instability, and epigenetic reprogram-ming in various cell types, tissues, and species, detectable even at environmentally and clinically relevant concentrations.

## 7. Neurotoxicity

In this chapter, we present a literature review of the neurotoxic effects of chlorpyrifos. Current knowledge in this area is derived from limited clinical data from human poisoning cases, as well as extensive experimental studies in animal models, particularly mammals. The mechanisms of action described to date rely largely on available in vitro research. Taken together, these studies enable the reconstruction of pathways underlying the neurotoxic effects of chlorpyrifos, including its impact on neurodevelopment, cognitive function, and neurodegenerative processes, and significantly expand our understanding of the potential consequences of human exposure to this compound.

Chlorpyrifos poses a significant neurotoxic risk to humans, with developing fetuses and children being particularly vulnerable. Neurotoxic effects of the pesticide have been observed even at low doses. Studies in young rats demonstrated that moderate inhibition of acetylcholinesterase (25–38%) can lead to long-lasting motor deficits, despite the absence of acute toxicity symptoms. Behavioral effects, such as reduced locomotor activity, appeared only after exposure had ended (postnatal days 25–30), when AChE activity was beginning to recover. These findings suggest the involvement of additional mechanisms contributing to neurological disturbances, including persistent alterations in cholinergic receptor density [140] and oxidative stress causing membrane lipid damage [141].

Like other organophosphates, chlorpyrifos is a potent generator of free radicals that can damage critical cellular components. The brain is particularly susceptible to oxidative damage due to its high content of unsaturated fatty acids, elevated oxygen demand, low regenerative capacity, and limited antioxidant defenses [142,143]. Studies have shown that chlorpyrifos-induced lipid peroxidation generates neurotoxic aldehydes such as 4-hydroxy-2-nonenal and malondialdehyde, which impair membrane integrity, disrupt synaptic function, and trigger neuroinflammation through microglial activation [144]. Prolonged oxidative stress consequently damages neuronal structures, especially in brain regions involved in memory and motor control, leading to neurocognitive deficits and the progression of neurodegenerative disorders [145,146,147].

In a mouse study by Deveci et al. (2018) [148], chlorpyrifos exposure was found to decrease total antioxidant capacity (TAC) and increase total oxidative capacity (TOC) in both plasma and brain tissue, further supporting its role in oxidative neurotoxicity. The literature data obtained from various experimental models (including PC12 cells as a model of sympathoadrenal neuronal cells, dopaminergic cell cultures, and in vitro studies in rats) indicate that oxidative stress induced by CP is closely associated with mitochondrial dysfunction [108,122,149,150,151].

Interestingly, research on chlorpyrifos has revealed toxicological effects comparable to those induced by certain chemical warfare agents, suggesting its potential involvement in the development of multisystem disorders observed in populations exposed to organophosphorus compounds, including Gulf War Illness (GWI), a chronic condition characterized by symptoms such as persistent fatigue, musculoskeletal pain, cognitive impairment, and gastrointestinal disturbances [152]. Animal studies have provided mechanistic insights into these effects. For instance, Locker et al. (2017) [153] demonstrated that chlorpyrifos-oxon induces pronounced neuroinflammatory responses in the brain, similar to those elicited by diisopropyl fluorophosphate (DFP). Notably, this pro-inflammatory activity was specific to organophosphorus compounds and did not correlate with acetylcholine levels, indicating that chlorpyrifos-oxon exerts its neurotoxic effects through pathways extending beyond cholinesterase inhibition, likely via direct activation of inflammatory signaling in glial cells [153].

Exposure to chlorpyrifos in Wistar rats resulted in marked oxidative and nitrosative stress, reflected by increased levels of reactive oxygen species (ROS) and nitric oxide (NO), decreased acetylcholinesterase activity, and loss of neurogenic cells within the amygdala. These molecular and cellular changes were accompanied by anxiety-like behaviors, including prolonged freezing, avoidance of the central zone in the open field test, and delayed transfer latency in the elevated plus maze [123].

Prolonged or subchronic exposure to chlorpyrifos disrupts the expression of neurotrophic factors and neuropeptides essential for neuronal survival, differentiation, and synaptic plasticity. Studies in rodents have shown that chlorpyrifos downregulates brain-derived neurotrophic factor (BDNF) and nerve growth factor (NGF), impairing neuroprotective pathways and neurotrophin-dependent synaptic maintenance [124,128,129,154,155]. Transcriptomic and immunohistochemical analyses demonstrated altered hippocampal expression of neuropeptide-related genes, including *Npy*, *Crhbp*, *Cort*, and *Pnoc*, accompanied by increased CREB phosphorylation and changes in miR-132/212 expression, which are associated with impaired learning and memory [124,128]. These transcriptional alterations likely result from disrupted cholinergic signaling, as neurotrophin gene expression is tightly regulated by muscarinic receptor activity, while neuropeptides may further enhance chlorpyrifos-induced neurotoxicity via G-protein-coupled pathways [154,156]. In further studies, Imam and colleagues confirmed that subchronic exposure to chlorpyrifos significantly triggered anxiety-like behaviors and working memory impairments in rats, which were correlated with oxidative damage, body weight loss, and a decreased number of proliferating cell nuclei in the hippocampus and amygdala [122].

Accumulating evidence indicates that CP-induced epigenetic alterations, encompassing changes in DNA methylation, histone modifications, and non-coding RNA expression, constitute an additional and significant dimension of its neurotoxicity, contributing to impaired neurodevelopment, disrupted neurotrophin signaling, and the progression of neurodegenerative processes. These mechanisms are discussed in detail in the preceding section [124,154,156].

Detoxification of hate pesticides, including CP, is primarily mediated by enzymes such as paraoxonase-1 (PON1) and carboxylesterase. Experimental studies in mice have demonstrated that deletion of the PON1 gene markedly increases sensitivity to the toxic effects of oxon metabolites, including chlorpyrifos-oxon and diazoxon [72,157]. The efficiency of detoxification is strongly influenced by PON1 polymorphisms: for example, the transgenic PON1 R192 variant provides greater protection against CP and CPO toxicity than the Q192 variant [158]. Similarly, carriers of low-activity variants such as PON1 55M or low-expression alleles like PON1-108T exhibit higher susceptibility to pesticide-related disorders, including PD [159] and brain tumors in children [160]. Low PON1 activity has been reported in neonates and infants, reaching adult levels only after 6–15 months, which may explain their increased vulnerability to organophosphate-induced neurotoxicity [161]. Epidemiological data confirm that reduced PON1 activity correlates with adverse developmental outcomes in exposed populations, such as shorter gestation, smaller head circumference, and impaired cognitive or psychomotor performance [162,163,164].

Clinically, patients with severe organophosphate poisoning present significantly lower serum PON1 activity compared with moderate cases, and enzyme levels correlate with both biochemical and clinical indicators of poisoning severity [21]. These findings underscore that diminished PON1 activity exacerbates oxidative stress and may serve as a prognostic marker of organophosphate toxicity.

Beyond acute poisoning, PON1 plays a critical role in susceptibility to neurodegenerative diseases such as AD and PD [165,166,167]. Environmental neurotoxins metabolized by PON1, including chlorpyrifos, may contribute to age-related neurodegeneration. Reduced PON1 expression and function promote oxidative and mitochondrial stress, the accumulation of toxic intermediates, and inflammation—processes believed to underlie neurodegenerative pathology [23,168,169]. Furthermore, studies indicate that reduced activity of specific PON1 polymorphisms may increase genetic susceptibility to PD [170,171], emphasizing the importance of PON1 genetic variability in modulating the neurotoxic effects of chlorpyrifos and other OPs [84].

Histopathological analyses by Deveci et al. further confirmed the neurodegenerative effects of CP in an experimental Parkinson’s disease model in Swiss albino mice. Repeated subcutaneous administration of chlorpyrifos-methyl induced marked neuronal degeneration and neurophagia in the substantia nigra, accompanied by the presence of intracytoplasmic, granular Lewy body-like inclusions, a pathological hallmark of PD. These changes were associated with reduced PON1 activity and TAC, increased TOC and total sialic acid (TSA) levels in brain tissue, indicating oxidative stress and mitochondrial dysfunction [148].

## 8. Neurodevelopmental Effects

Chlorpyrifos easily crosses the placental barrier, affecting fetal development, including the nervous system. Studies on animal models indicate that exposure to CP can lead to impaired cognitive, motor, and behavioral functions in rodents [163,164,165,166,167]. Additionally, male mice exposed to chlorpyrifos showed dose-dependent muscle fiber degeneration and impaired sperm parameters, resulting in adverse effects on pregnancy outcomes [172].

Epidemiological analyses have shown that prenatal exposure to CP is associated with developmental neurotoxicity (DNT), the effects of which can manifest in later childhood, leading to learning disabilities, attention deficit hyperactivity disorder (ADHD), as well as reduced IQ and working memory [56,173,174].

Chlorpyrifos may influence the regulation of peroxisome proliferator-activated receptor gamma (PPARγ), a transcription factor essential for placental development, energy metabolism, and the regulation of inflammatory and immune processes [175], as well as early brain formation [176]. Emerging evidence suggests that chlorpyrifos exposure can induce epigenetic modifications of *PPARγ*, including DNA methylation within the promoter region of the *PPARγ* gene and trimethylation of histone H3 at lysine 4 (H3K4me3) [177,178]. These modifications lead to decreased PPARγ expression, which has been linked to poorer cognitive and language outcomes in two-year-old children, particularly in boys [177]. Consistent with these findings, in vitro studies using the human SH-SY5Y cell line showed that chlorpyrifos treatment reduced PPARγ expression through DNA methylation of the *PPARγ* promoter region [178].

In addition to DNA methylation, chlorpyrifos exposure has been shown to induce specific histone modifications in human neural progenitor cells (hNPCs). Kim et al. (2016) [125] demonstrated that chlorpyrifos increased phosphorylation of histone H3 at serine 10 (H3S10ph) and dimethylation at lysine 4 (H3K4me2) under proliferative conditions, while significantly reducing histone deacetylase 4 (HDAC4) expression under differentiation conditions. These findings indicate that chlorpyrifos can alter chromatin remodeling in a differentiation stage-dependent manner, potentially affecting gene expression and neuronal development. Moreover, chlorpyrifos affected the expression of key proteins involved in neuronal proliferation and differentiation. Specifically, changes were observed in β-tubulin III, SOX2, PCNA, and MAPK signaling pathways, indicating that chlorpyrifos may interfere with neural progenitor cell maturation and survival [125].

Chlorpyrifos has been shown to adversely affect the developing brain by interfering with the expression of key neurotrophic factors in the Fibroblast Growth Factor (FGF) family, particularly *FGF2* and *FGF20* which are crucial for the proper development of specific brain regions. *FGF2* plays a critical role in the development of the hippocampus, a region essential for memory and learning, while *FGF20* is vital for the striatum, a brain structure involved in motor control, motivation, and cognitive processes. Downregulation of *FGF2* in the brain stem and *FGF20* in the forebrain, along with dysregulation of *FGFR4* and *FGF22*, has been linked to cognitive impairment in experimental animal models [179].

Studies showed that the neonatal brain is particularly vulnerable to CP and this mechanism of neurotoxicity is partially independent of CP’s ability to inhibit cholinesterase and exhibits regional selectivity. This implies that even exposure levels to CP insufficient to significantly inhibit cholinesterase can still cause harmful changes in the brain by disrupting FGF signaling. These changes are related to *FGF2* and *FGF20*, which play protective roles by shielding dopaminergic neurons from oxidative damage, aiding in their repair [180,181]. Studies showed that, deficiencies in *FGF2* have also been identified in dopaminergic neurons associated with PD [182]. Early-life exposure to chlorpyrifos may contribute to an increased risk of PD later in life by disrupting FGF2 and FGF20 signaling [179].

To eliminate maternal factors inherent in mammalian models, Slotkin et al. conducted studies using fertilized chicken eggs. Chlorpyrifos was injected at doses of 10 or 20 mg/kg on days 2 and 6 of incubation. After hatching, markers of acetylcholine and serotonin (5-HT) systems were evaluated. The higher dose CP resulted in a notable decrease in cholinesterase activity, while both doses significantly reduced the presynaptic high-affinity choline transporter. Additionally, CP exposure decreased the expression of 5HT1A receptors in the cerebral cortex. These findings mirror those observed in rodent models, highlighting the direct neurotoxic effects of chlorpyrifos on the developing brain, particularly in disrupting the maturation of cholinergic and serotonergic pathways [183].

In vitro studies on neurons and glial cells have demonstrated that metabolite of chlorpyrifos, chlorpyrifos-oxon can also inhibit neurite outgrowth, disrupting the formation of neuronal connections. Experiments with primary neurons revealed that CPO, even at concentrations lower than those required to inhibit AChE activity, induces a more than 3-fold increase in phosphorylation level of CREB protein in cultured neurons associated with cell development [184,185].

Moreover, research indicates that chlorpyrifos-oxon disrupts neuronal development by interfering with cytoskeletal integrity. It forms covalent adducts with tubulin, leading to its phosphorylation, aggregation, and loss of microtubule stability [186,187]. In differentiating N2a neuroblastoma cells, sub-lethal CPO concentrations (1–10 μM) inhibited neurite outgrowth and reduced the levels of key axonal proteins such as GAP-43 and neurofilament heavy chain [188]. These effects occurred independently of marked AChE inhibition, indicating alternative mechanisms of neurotoxicity. Collectively, the findings suggest that CPO impairs axonal growth and cytoskeletal organization essential for neuronal differentiation and synapse formation. In addition, this compound has been shown to interact with muscarinic receptors (m_2_ and m_4_ subtypes) and to inhibit adenylate cyclase activity in rat striatal tissue as well as in NG108-15 neuroblastoma-glioma and CHO cells expressing human muscarinic receptors. Notably, this inhibition was atropine-insensitive and persisted in pertussis toxin–treated cells, suggesting that CPO suppresses adenylate cyclase through a receptor-independent mechanism, likely via direct interaction with the enzyme itself [189]. Importantly, this effect is as much as nine times stronger in newborns than in adult rats, suggesting greater sensitivity of the developing brain to the effects of CPO [190].

Moreover, chlorpyrifos-oxon interacts with nicotinic, cannabinoid CB1 receptors [191,192], and also directly phosphorylates muscarinic receptors in cardiac tissue, disrupting signal transduction independently of AChE inhibition [193,194]. CPO has been shown to potently inhibit CB1 receptor binding in mouse brain at concentrations as low as 14 nM, indicating a strong potential to interfere with endocannabinoid signaling [195]. Additionally, CPO inhibits key enzymes of the endocannabinoid system, such as monoacylglycerol lipase (MAGL) and fatty acid amide hydrolase (FAAH), leading to impaired degradation of 2-AG and anandamide and subsequent dysregulation of CB1-mediated signaling in the developing brain, which may result in long-term behavioral deficits [196,197]. Furthermore, CPO forms covalent adducts with albumin and other tyrosine-containing proteins, as evidenced by the detection of diethoxyphosphorylated tyrosine up to five days after exposure [23,198]. These modifications may impair protein function and contribute to prolonged toxicokinetics and systemic distribution of CPO, as well as adversely affect neurodevelopment [23,195,199]. Moreover, CPO’s strong affinity for NMDA receptors suggests it may disrupt glutamatergic neurotransmission, potentially contributing to its neurotoxic effects. These findings complement earlier observations of CPO’s actions on cholinergic and endocannabinoid systems, highlighting its multi-target mechanism of toxicity, potentially contributing to the neurotoxic effects [191].

The ability of CPO to inhibit adenylate cyclase and diacylglycerol lipase activity in glial and neuronal cells disrupts responses to growth signals, further impairing developmental processes [189]. Beyond this, the study by Bomser et al. (2002) [200] demonstrated that CPO activates ERK 44/42 signaling in CHOK1 cells by inhibiting diacylglycerol (DAG) lipase, leading to DAG accumulation and activation of PKC/MAPK signaling. This represents a distinct mechanism of toxicity that is independent of AChE inhibition. The findings reveal an alternative toxicity pathway where CPO potentiates cellular responses by disrupting lipid messenger metabolism [200].

In brain cell cultures derived from the fetal rat telencephalon, CPO was shown to reduce the activities of choline acetyltransferase and glutamate decarboxylase with at least 100-fold greater potency than chlorpyrifos, its parent compound [201]. Additionally, this metabolite is approximately 3 times more potent than CP in activating mammalian signal transduction pathway ERK1/2. This pathway is involved in regulation of synaptic plasticity, brain development and repair as well as memory formation. This pathway is also a potent effector of neuronal death and neuroinflammation in many CNS diseases [89,202,203]. Overall, the ability of CPO to interfere with normal developmental processes in the nervous system far exceeds that of its parent compound [184].

The CELSPAC-SPECIMEn study analyzed urinary levels of chlorpyrifos and other pesticides in a cohort of Czech adults and children to assess exposure patterns. The highest concentrations of CP were found in children, and significant seasonal variations were observed, with metabolite levels being significantly higher during winter compared to summer [204]. The study also evaluated the impact of CP and other pesticides on the levels of epigenetic markers, including DNA methylation levels and oxidative stress markers (8-hydroxydeoxyguanosine, 8-OHdG). The presence of chlorpyrifos metabolites was correlated with increased urinary biomarkers of cytosine methylation-5-methylcytosine (5-mC) and 5-methyl-2′-deoxycytidine (5-mdC), indicating that exposure has a negative impact on DNA methylation patterns in humans [204].

In turn, in other studies an increase in urinary 8-OHdG levels was observed as early as the first day after chlorpyrifos spraying in farmers [205], while children from agricultural areas exhibited lower glutathione levels, a key component of the antioxidant system, compared to their urban counterparts [57]. Additionally, the presence was confirmed of chlorpyrifos metabolites correlated with increased urinary biomarkers of cytosine methylation in children, highlighting potential negative impacts of chlorpyrifos exposure on methylation patterns in human biomonitoring data [206].

Research on pesticides, such as chlorpyrifos and its metabolites reveals notable discrepancies between independent studies and those funded by the industry. These differences may partly reflect variations in how data are selected and evaluated in regulatory risk assessments. Only a small fraction (13–15%) of published academic toxicity studies are typically incorporated into pre-market assessments, while industry-standard test guidelines are often preferentially applied, despite evidence suggesting they may be less sensitive to low-dose effects [207]. A key example is the 1972 safety evaluation for chlorpyrifos (the so-called Coulston study), funded by Dow Chemical, which for decades served as the basis for the EPA to establish a NOAEL of 0.03 mg/kg/day. Sheppard et al. (2020) [208] reported that the study was not peer-reviewed, and their independent re-analysis suggested that certain baseline data may not have been included in the original statistical evaluation. Incorporating these data was estimated to lower the safe threshold to at least 0.014 mg/kg/day. Not until 1998 was the reference dose reduced to 0.0003 mg/kg/day to comply with requirements for protecting children’s health, and current stringent standards are significantly lower [208]. Independent research indicates a potentially higher risk to human health, particularly for children, due to exposure to this pesticide. Chlorpyrifos exposure has been linked to permanent neurological damage and reduced IQ in children [209]. Consequently, both Sheppard et al. (2020) and Mie and Ruden (2018) emphasize the urgent need for an independent review of all registrant-sponsored studies used in setting regulatory standards [208,209]. The societal costs associated with these risks are substantial, highlighting the urgent need for stricter regulations on chlorpyrifos use [56,209].

## 9. Endocrine-Disrupting Effects

Chlorpyrifos is recognized as an endocrine disruptor (ED), interfering with hormonal homeostasis at multiple levels and affecting enzymatic pathways involved in hormone biosynthesis or metabolism [6,210,211]. Research has demonstrated that developmental exposure to low doses of CP in mice alters thyroid function and disrupts thyroid hormone levels [212]. Consistently, experimental studies have shown that long-term exposure to CP, from prenatal stages through adulthood, induces hypothyroidism and anti-androgenic effects in male rats, accompanied by morphological alterations in the adrenal and thyroid glands, as well as reduced sperm count and prostate weight [213]. These anti-androgenic activities are further supported by evidence that CP exposure impairs male reproductive performance by altering the expression of critical steroidogenic enzymes [214]. Moreover, both developmental and lifelong exposure to CP disrupts thyroid hormone signaling in the liver, resulting in sex- and generation-specific changes in glucose homeostasis via activation of the triiodothyronine–forkhead box O1 (T3–FOXO1) axis [215].

Recent molecular evidence from both in vitro (mHypoE-N46 cells) and in vivo (mouse models) studies has demonstrated that CP exposure significantly upregulates the expression and secretion of key orexigenic neuropeptides, specifically Neuropeptide Y (Npy) and Agouti-related peptide (Agrp). Notably, this disruption is characterized by a selective upregulation of estrogen receptor beta (ERβ) and an increased ERβ/ERα ratio in the hypothalamus, with pharmacological inhibition confirming ERβ as the primary mediator of CP-induced effects. Furthermore, the observed increase in leptin receptor (Lepr) expression suggests that CP may fundamentally recalibrate the hypothalamic circuitry governing energy homeostasis and appetite regulation [216]. Additional studies in mice have demonstrated that low-dose CP exposure promotes obesity and metabolic dysfunction [217,218]. In adulthood, CP exposure induces an obesogenic and diabetogenic phenotype in apoE3 mice, characterized by excessive weight gain associated with increased food intake, as well as elevated levels of glucose, insulin, and total cholesterol [217]. Similarly, dietary CP exposure enhances weight gain in mice fed a high-fat diet under thermoneutral conditions, likely through impaired mitochondrial function and reduced thermogenesis in brown adipose tissue [218]. Additionally, other researchers have demonstrated that exposure to ecologically relevant concentrations of the CP disrupts sex differentiation and reproductive development in *Rana dalmatina* through endocrine-mediated pathways [219]. These findings are further supported by biomarker-based studies in marine organisms which have shown that sublethal exposure to chlorpyrifos can interfere with estrogen signaling, highlighting its endocrine-disrupting potential across diverse species [220].

Furthermore, Fouyet et al. (2022) [221] demonstrated that CP acts as a placental endocrine disruptor by altering hormone secretion and activating the P2X7 death receptor, a key biomarker of endocrine toxicity for pregnant women. Interestingly, their findings suggest a ‘reverse cocktail effect,’ where the presence of CP in lavender essential oils may partially mask its pro-apoptotic signaling while still maintaining its primary endocrine-disrupting activity [221].

Molecular docking studies provide strong structural evidence that chlorpyrifos and its environmental degradation products interact directly with human estrogen receptors (ER), identifying specific amino acid residues responsible for these binding events. These interactions trigger conformational changes in the ER that disrupt estrogen signaling, potentially leading to impaired reproductive health and the proliferation of mammary tissue [222].

## 10. Liver Damage and Disruption of Gut Microbiota Balance

Exposure to chlorpyrifos is increasingly recognized as a significant contributor to chronic inflammation and liver injury. In addition to its direct hepatotoxic effects, chlorpyrifos disrupts the gut microbiota, triggering secondary mechanisms of liver damage via the gut–liver axis. This dual pathway, comprising both direct toxicity and microbiota-mediated injury, underscores the systemic complexity of chlorpyrifos exposure and highlights the liver as a primary target of its harmful effects [223,224,225]. Zhang et al. (2021) [226] in studies in mice have demonstrated dose-dependent liver tissue changes following CP exposure. At doses of 1 mg/kg and 10 mg/kg, focal inflammation and hepatocyte necrosis were observed. These pathological changes correlated with increased levels of pro-inflammatory cytokines, such as TNF-α, IL-1β, and IL-6, which are hallmark markers of liver inflammation [226].

In mice exposed to chlorpyrifos, beneficial bacteria such as *Akkermansia* and *Butyricimonas* were reduced, while pathogenic bacteria, including Helicobacter and Desulfovibrio, proliferated. The decline in *Akkermansia* is particularly concerning, as this genus plays a critical role in maintaining intestinal barrier integrity. Disruptions to the microbiota increase intestinal permeability, facilitating the translocation of bacteria and their metabolites to the liver, thereby triggering inflammatory responses [226]. Studies by Zhao et al. (2016) demonstrated that exposure to chlorpyrifos significantly disrupts the balance of the gut microbiome, reducing the abundance of beneficial bacteria from the *Lactobacillus* and *Bifidobacterium* genera while simultaneously increasing populations of *Firmicutes*, *Bacteroidetes* and *Proteobacteria* [227]. Furthermore, a study by Walker et al. (2011) confirmed significant association between changes in the proportions of *Firmicutes* and *Bacteroidetes* and chronic intestinal inflammation [228]. The study by Song et al. (2024) [223] further supports evidence that CP induces liver damage through alterations of the gut microbiota. In a mouse model, it was demonstrated that chronic exposure to a low dose of CP leads to pathological alterations in hepatic tissue and functional impairments, as evidenced by increased serum levels of alanine aminotransferase (ALT) and total bile acids. Pretreatment with antibiotics, which alters the composition of the gut microbiota effectively attenuated CP-induced liver damage. Microbiological analysis indicated that specific groups of gut bacteria, such as *Saccharibacteria*, *Odoribacter*, *Enterococcus*, and the genus AF12, have a potential role in modulating CP hepatotoxicity [223]. Similar conclusions were drawn in another study demonstrating that environmentally relevant doses of chlorpyrifos can directly alter the metabolic activity of gut microbiota. Although CP did not exert a direct bactericidal effect, it significantly perturbed microbial metabolism, leading to increased concentrations of amino acids, carbohydrates, and nucleic acids. Moreover, exposure to CP was associated with shifts in the relative abundance of several bacterial genera, including *Lactobacillus*, *Allobaculum*, *Roseburia*, and *Butyricicoccus*. Functional predictions based on 16S rRNA sequencing indicated reduced amino acid biosynthesis and nucleic acid degradation alongside enhanced glycolytic activity, suggesting that CP exposure may disrupt gut metabolic homeostasis and thereby contribute to host metabolic imbalance [229]. Similar conclusions were reached by Durairaj et al. (2025) [230], who demonstrated that chronic exposure to human-equivalent doses of CP triggers hyperglycemia by inducing gut dysbiosis. This mechanism involves the depletion of beneficial taxa, such as *Lactobacillus* and *Akkermansia*, alongside an enrichment of pathobionts including *Helicobacter* and *Alistipes*, correlating with the incidence of type 2 diabetes in non-obese populations [230].

In the case of the zebrafish (*Danio rerio*) model organism, similar metabolic disruptions have been observed, providing a valuable comparative perspective on the gut–liver axis. The research by Wang et al. (2019) [231] demonstrates that after 21 days of exposure to CP (30, 100, 300 μg/L), *Danio rerio* exhibited oxidative stress, gut microbiota dysbiosis, and liver metabolic disturbances. However, malondialdehyde (MDA) levels increased, while glutathione (GSH) content decreased in the gut in these model organism. A significantly altered *Proteobacteria* abundance and 25 other bacterial genera was also observed. GC-MS analysis revealed that CP influenced 98 liver metabolites, linked to glucose and lipid metabolism, the TCA cycle, and amino acid metabolism. Moreover, CP exposure downregulated genes related to glycolysis and lipid metabolism suggests liver metabolic disruption. However, when interpreting these results, it is essential to acknowledge the significant physiological differences between teleosts and mammals. Zebrafish possess a simplified hepatic structure and a gut microbiome typically dominated by *Proteobacteria*, in contrast to the *Firmicutes* and *Bacteroidetes* prevalent in humans. Despite these differences, the fundamental pathways of CP-induced toxicity appear to be conserved across species [231].

The mechanism responsible for the damage to the intestinal barrier induced by chlorpyrifos involves impairment of zonula occludens-1 (ZO-1), a key protein essential for maintaining tight junctions between intestinal cells. Their destabilization leads to increased intestinal permeability. Bacterial DNA was detected in the liver tissue of mice exposed to high doses of CP providing evidence of bacterial translocation from the intestine to the liver as a consequence of the compromised intestinal barrier [232,233].

Liang et al. (2019) [234] confirmed in a mouse model that CP exposure contributes to hepatic inflammation, as evidenced by increased expression of TLR-4 and pro-inflammatory cytokines such as TNF-α in the liver. Furthermore, this study confirmed that chlorpyrifos significantly reduces the expression of genes encoding tight junction proteins (occludin, claudin-1, and ZO-1), thereby increasing intestinal permeability. This disruption of tight junction proteins level promotes lipopolysaccharide (LPS) translocation into the circulation, triggering low-grade systemic inflammation and ultimately leading to liver damage, obesity and insulin resistance [234].

Fu et al. (2024) [235] investigated the hepatotoxic effects of CP in rats exposed to low, medium, and high doses (2.5, 5, and 10 mg/kg body weight). The study demonstrated that CP exposure impaired liver function by inducing oxidative stress, which in turn exacerbated inflammation and apoptosis. Activation of the JAK/STAT and MAPK signaling pathways was observed, accompanied by increased expression of inflammatory markers (IL-1β, IL-6, TNF-α) and pro-apoptotic proteins (Bax, Caspase-3, cPARP1). According to Fu et al. (2024), these pathways act as central mediators of CP-induced hepatotoxicity and represent potential molecular targets for mitigating chlorpyrifos-related liver injury [235].

Research by Han et al. (2023) [236] showed that chlorpyrifos promotes ferroptosis in hepatocytes through a GSDMD-dependent mechanism. In AML12 cells, CP (LD_50_ = 50 μM) triggered ferroptotic death characterized by excessive mitochondrial ROS formation (mtROS), iron accumulation, and oxidative imbalance (↓SOD/GSH-Px, ↑MDA). Mechanistically, CP binds to GSDMD at Ser234, inducing its cleavage into NT-GSDMD, which damages mitochondria and increases mtROS leakage. CP simultaneously activates p53, creating a feed-forward loop that intensifies ferroptosis. Knockout of GSDMD or p53, and treatment with ROS inhibitor YCG063 or ferroptosis suppressor Fer-1, significantly reduced CP toxicity. In vivo studies in GSDMD^−^/^−^ mice and Fer-1-treated wild-type mice confirmed that the GSDMD–mtROS–p53 axis drives CP-induced liver injury [236].

As demonstrated by Montanari et al. (2024) [94] acute exposure of HepG2 cells to sub-toxic doses of chlorpyrifos significantly increased intracellular ROS and mitochondrial superoxide production, leading to a decrease in mitochondrial membrane potential (ΔΨm). These effects indicate a direct impairment of mitochondrial activity and bioenergetics, primarily due to ROS overproduction, including mitochondrial superoxide and hydrogen peroxide [94].

Evidence also indicates that exposure to chlorpyrifos inhibits PON1 activity, potentially linking it to liver damage [148]. PON1, an antioxidant enzyme associated with high-density lipoprotein (HDL) proteins such as apo A1 and apo J (clusterin), degrades oxidized lipids and prevents the accumulation of lipid peroxides in both HDL and low-density lipoprotein (LDL) fractions [237,238]. Organophosphate pesticides, including CP, can also disrupt lipid profiles by increasing total and LDL cholesterol while reducing HDL cholesterol [239]. Analyses by Deveci et al. (2018) [148] revealed a correlation between low HDL levels, elevated LDL levels, and lipid oxidation processes in CP-exposed mice. Furthermore, the increased total cholesterol observed in the CP group may reflect cholestasis and hepatocellular injury induced by chlorpyrifos exposure [148].

## 11. Musculoskeletal Disorders

Increasing evidence indicates that exposure to chlorpyrifos leads not only to damage to the central nervous system, endocrine disruption, gut microbiota dysbiosis, and hepatotoxicity, but also exerts a significant toxic impact on bone metabolism. Experimental studies have shown that exposure to this pesticide can induce bone loss, particularly in trabecular bone, through direct toxic effects on osteoblasts and osteoclasts, as well as through the induction of oxidative stress in bone tissue [240,241,242].

Consistent with these observations, studies by Ali et al. demonstrated that chlorpyrifos markedly disrupted trabecular bone microarchitecture in mice, resulting in reduced bone volume (BV/TV), bone mineral density (BMD), and trabecular number (Tb.N), along with increased trabecular separation (Tb.Sp) and structural model index (SMI) [243]. CP reduced the expression of key osteogenic markers, including *Runx2*, *Col1a1*, and *Atf4*, thereby impairing the bone formation, while simultaneously increasing the expression of the osteoclastogenic marker Trap, promoting bone resorption. An increase in the number of active osteoclasts and their resorption surface area relative to bone surface (Oc.S/BS) was observed. Importantly, these skeletal alterations occurred alongside Parkinsonian-like behavioral changes, reduced dopamine and acetylcholinesterase activity in the striatum, and the loss of dopaminergic neurons in the nigrostriatal pathway, indicating that CP-induced bone deterioration develops in parallel with broader neurotoxic effects in this exposure model [243].

The literature data indicate that chlorpyrifos can damage the diaphragm, a key skeletal muscle essential for respiratory processes during the inspiratory phase. The most significant alterations were observed in the structure of this organ and its biochemical balance. Chlorpyrifos exposure modified the diaphragm structure and biochemical balance, including reduced cholinesterase activity, and can damage the diaphragm [244,245,246,247]. In a study conducted by Sabbouri et al. (2022) [248] on the chronic exposure of adult rats to CP, a significant increase in diaphragm contractile tension in a dose-dependent manner was observed. The CP1 group (1 mg/kg/day) exhibited a 130% increase, while the CP5 group (5 mg/kg/day) showed a 96% increase compared to the control group. Both groups also exhibited prolonged time to reach maximum tension and an extended half-relaxation time, indicating altered muscle contractile properties [248]. Additionally, chronic exposure to CP led to a significant decrease in serum testosterone levels in both CP1 and CP5 groups compared to the control group. Testosterone plays a critical role in muscle maintenance and function, including resistance to fatigue and structural integrity. Its reduction has been linked to impaired muscle endurance and increased fatigability, which could further exacerbate diaphragm dysfunction under CP exposure [248,249]. At the same time, elevated levels of corticosterone and growth hormone were detected, which may represent an adaptive response to oxidative stress induced by CP exposure. In the CP5 group, the observed increase in myofibrillar protein content could be related to enhanced contractile activity of the diaphragm [248]. Changes in myosin heavy chain (MHC) isoform expression were also observed. In the CP1 group, MHC I expression decreased significantly by 31%, while MHC IIa expression increased by 14%, suggesting a shift toward a more fatigable muscle phenotype. In contrast, the CP5 group showed no significant differences in MHC isoform expression compared with the control, although a slight, non-significant increase in MHC I expression was noted. These findings suggest that the effects of CP on myofibrillar protein composition and muscle contractility are dose-dependent and may contribute to altered diaphragm function [248].

Another study demonstrated that prolonged exposure to chlorpyrifos alters the contractility of both slow-twitch soleus muscles and fast-twitch extensor digitorum longus (EDL) muscles in adult rats, indicating its widespread impact on skeletal muscle function [250]. Similarly, Sekaran et al. (2023) [251] investigated the effects of chlorpyrifos and its metabolite, 3,5,6-trichloro-2-pyridinol (TCP), on the skeletal system of chick embryos. Their findings revealed that both the pesticide and its metabolite disrupt chondrogenesis within the cartilage of the growth plate in long bones and interfere with the ossification process. Furthermore, a significant reduction in the expression of essential transcription factors, including SOX9, RUNX2, and ALP, was observed in chick embryos, highlighting their involvement in skeletal developmental disorders. The downregulation of these factors suggests that chlorpyrifos exposure may impair skeletal development by disrupting the transcriptional network essential for endochondral ossification [251].

## 12. Cancers

Chlorpyrifos exhibits potential pro-carcinogenic activity through mechanisms involving oxidative stress, DNA damage and impaired repair, as well as epigenetic alterations. CP exposure has been associated with hepatic, breast, and ovarian tumorigenesis [252]. In the study by Balakrishnan et al. (2025) [252] using the human fetal liver epithelial cell line (WRL-68), CP downregulated PON1, leading to intracellular accumulation of the pesticide and its metabolites and resulting in increased cellular damage. CP also disrupted DNA repair pathways by upregulating *BRCA1*, *BRCA2*, and *RB1* while downregulating *TP53* and *PARP1* [252].

Significant epigenetic effects were also observed. CP reduced the de novo expression of the methyltransferases DNMT3A and DNMT3B, causing global hypomethylation. Global DNA hypomethylation is generally associated with increased genomic instability, which subsequently leads to the activation of oncogenes and the dysregulation of genes involved in cell cycle control and DNA damage response, thereby increasing the risk of neoplastic transformation of normal cells. These changes were correlated with increased expression of Cdc25a, indicating enhanced proliferation. CP further influenced the epithelial–mesenchymal transition by reducing E-cadherin expression and increasing N-cadherin expression, as well as increasing the expression of MMP-2 and MMP-9, which facilitate extracellular matrix degradation and may increase metastatic potential. Prolonged exposure to CP further reinforced these molecular alterations, exacerbating genetic instability and cell cycle dysregulation. Collectively, these findings support the role of CP in initiating and promoting malignant transformation in hepatocytes [252].

CP and its metabolite, CPO also exhibit cytotoxic effects in liver cancer cells, potential impact on drug resistance, detoxification, and liver carcinogenesis mechanisms. In HepG2 cells, CP exposure resulted in significantly higher expression of drug efflux transporters (P-glycoprotein, BCRP), metabolic enzymes (CES2, PON1), and nuclear receptors (AhR, PXR), as well as the transcription factor Nrf2, compared with kidney epithelial HK-2 cells. These changes may underlie liver-specific toxicity and contribute to potential carcinogenic mechanisms associated with CP exposure [253].

In vivo studies have shown that chronic exposure to chlorpyrifos, even at subtoxic doses, increases the risk of breast cancer by shortening tumor latency and increasing the number of tumor lesions, likely due to its endocrine-disrupting properties. Complementary in vitro experiments demonstrated that CP stimulates the proliferation of estrogen-dependent MCF-7 breast cancer cells through activation of the estrogen receptor alpha signaling pathway [254].

Ventura et al. (2016) [210] confirmed the endocrine-disrupting effects of CP in the mammary glands of rats. CP increased the expression of the progesterone receptor, while simultaneously decreasing the levels of estrogen receptor activity repressors (REA and SMRT) and phosphorylation of estrogen receptor (pERα-Y537), suggesting potential estrogen-like activity. High-dose CP exposure led to a marked reduction in circulating estradiol and progesterone levels, and also a strong suppression of luteinizing (LH) hormone levels at both low and high doses, while the follicle-stimulating hormone remained unaffected. The suppression of LH persisted even in ovariectomized rats, suggesting that CP can mimic estrogen-like negative feedback on the hypothalamic-pituitary-gonadal axis. In a rat model, CP significantly increased the number of ducts in mammary tissue and elevated the incidence of florid ductal hyperplasia. These histological changes were accompanied by a rise in lobular adenosis, particularly in both non-sclerosing and sclerosing forms, with the effects most pronounced at the lower dose. In addition, enhanced cell proliferation was confirmed by immunohistochemistry, as evidenced by increased PCNA expression. These findings underscore the detrimental effects of CP on the mammary gland and suggest that the associated hormonal imbalances and molecular alterations may contribute to an increased risk of hormone-dependent cancers, particularly breast cancer [210].

Further studies have shown that CP modulates estrogen and progesterone receptor activity through epigenetic regulatory enzymes. In mammary gland tissues, CP exposure has been shown to upregulate the expression of the histone deacetylase HDAC1, which may be recruited to the estrogen receptor α (ERα) promoter region, resulting in transcriptional repression and increased clonogenic potential. These findings suggest a role for CP in promoting tumorigenic phenotypes [255].

Lasagna et al. (2022) [211] reported that chlorpyrifos, as an endocrine disruptor, promotes the progression of more aggressive and metastatic forms of breast cancer. Studies using 3D models demonstrated that even at low concentrations (0.05 μM), chlorpyrifos induces breast cancer cell migration and invasion by activating ERα and c-SRC receptors, leading to the phosphorylation of AKT and GSK-3β. At higher concentrations (50 μM), an increase in p38 phosphorylation through the c-SRC pathway was also observed [211].

Chlorpyrifos is known to exhibit estrogenic properties, and its ability to mimic or disrupt hormonal signaling may contribute to the proliferation of the ovarian surface epithelium, which widely considered the primary site for the onset of ovarian cancer. CP exposure has been shown to disrupt the estrous cycle in rats, particularly by prolonging the metestrous phase, and to induce notable histological changes, including increased thickness of the uterine surface epithelium and the myometrium. Additionally, structural abnormalities such as degeneration of oocytes and nuclear damage in ovarian cells have been documented following CP exposure, suggesting a potential for genomic instability within five tissues that may facilitate malignant transformation [77,256,257,258].

Additionally, studies suggest that chlorpyrifos may contribute to cancer initiation and progression through inflammatory mechanisms, including COX-2 upregulation, and by promoting angiogenesis via HIF-1α and VEGF-A signaling in breast cancer cells. In these cells, CP has been shown to disrupt key cellular pathways, such as the aryl hydrocarbon receptor (AhR) and NO-mediated signaling [259]. Similarly, Zárate et al. (2020) confirmed that CP exposure stimulates angiogenesis in MCF-7 breast cancer cells, an effect associated with increased NO production, and elevated VEGF-A and COX-2 expression [260].

Alternative splice variants of AChE, such as AChE-S, play important roles not only in synaptic function but have also been implicated in cell cycle regulation (cyclin D1, c-Myc) and proliferation via the Wnt/β-catenin pathway. Studies showed that CP treatment resulted in a concentration-dependent increase in AChE-R variant expression after both one and fourteen days of treatment, whereas AChE-S is induced only at higher concentrations (10–100 µM). Low doses of CP (0.1–1 µM) enhance cell proliferation by activating the Wnt/β-catenin signaling pathway, increasing the expression of β-catenin, c-Myc, and cyclin D1, while also inhibiting GSK-3β activity [261]. In contrast, high concentrations of chlorpyrifos enhance GSK-3β activity, leading to β-catenin degradation and a reduced expression of its downstream oncogenes, suggesting that CP may interfere with tumor-promoting signaling and influence cancer cell proliferation and survival [261]. Furthermore, CP exposure has been showed to reduce cell viability [262,263], likely due to oxidative stress induction, lipid peroxidation and overexpression of AChE-S variant and can even induce cell cycle arrest [254,264], highlighting its cytotoxic effects in breast cancer cells [264].

Studies have demonstrated that CP exposure leads to increased reactive oxygen species and lipid peroxidation products, such as malondialdehyde (MDA), in MCF-7 and MDA-MB-231 cells. This oxidative response was accompanied by downregulation of NRF2 and HO-1, key regulators of the cellular antioxidant defense system. While acute oxidative stress can compromise cell viability, chronic exposure to moderate ROS levels has been associated with tumor progression and therapy resistance. Exposure to 50 μM CP has also been shown to induce a rapid increase in ROS generation as early as 10 min after treatment [261].

Ventura et al. (2015) [265] demonstrated that CP-induced increases in H_2_O_2_ levels lead to phosphorylation of ERK1/2, resulting in inhibition of cell proliferation and promotion cell death. Elevated ROS levels in cells can impact redox-sensitive molecules, potentially driving proliferation, differentiation, altered response to anticancer therapies, and inducing mutations or genomic instability—suggesting that the surviving cells might initiate carcinogenic processes [265].

Epidemiological evidence supports a link between CP exposure and breast cancer risk. Studies has shown that women exposed to CP face a higher risk of developing breast cancer compared to unexposed individuals. This conclusion is based on findings from the Agricultural Health Study, a large prospective cohort involving 30,003 women—spouses of pesticide applicators—who provided self-reported data on their lifetime use of organophosphate pesticides, including CP. The study identified a general association between OPs exposure and increased breast cancer risk (RR = 1.20, 95% CI: 1.01–1.43), with an even higher risk observed among postmenopausal women (RR = 1.27, 95% CI: 1.00–1.62). Notably, chlorpyrifos exposure was significantly associated with an increased risk of developing estrogen receptor-negative and progesterone receptor-negative (ER-PR-) breast cancer (RR = 2.26, 95% CI: 1.07–4.75) [266].

Evidence from biomarker studies further supports the link between chlorpyrifos exposure and oxidative stress. Wang et al. (2016) [267] provided compelling evidence of oxidative stress in individuals exposed to chlorpyrifos during agricultural activities. Elevated levels of 8-OHdG, a well-established biomarker of oxidative DNA damage, were detected in the urine of farmers following chlorpyrifos application. Urine samples also contained the metabolite TCP, confirming absorption and biodistribution of the pesticide. The persistence of this metabolite for several days post-exposure highlights the compound’s bioavailability and potential for bioaccumulation. These results indicate that exposure to chlorpyrifos can lead to increased oxidative damage at the cellular level, potentially contributing to mutagenesis and long-term health risks [267].

## 13. Conclusions and Future Perspectives

The widespread use of chlorpyrifos presents a significant challenge to public health and environmental sustainability. While it was once a dominant agricultural tool, mounting evidence of its risks has led to a varied regulatory status worldwide, ranging from complete bans in the European Union to increasing restrictions in the United States and specific permitted applications in regions such as India and Egypt. Despite these regulatory actions, chlorpyrifos residues and their metabolites persist globally and remain detectable in soil, natural waters and food (fruits, cereals, and vegetables), as well as in human tissue and urine samples [7].

To safeguard both human health and the environment, future efforts should prioritize:

(1) Improving of regulatory frameworks: An objective risk assessment requires the integration of independent academic data alongside findings from industry-sponsored studies [7,207]. Differences between studies arising from variations in experimental protocols should also be taken into account, as they may affect data interpretation and the robustness of risk assessment [209]. The application of advanced statistical methods, including multi-level modeling such as linear mixed-effects models, may improve the analysis of variability in chlorpyrifos exposure and facilitate the identification of factors contributing to these differences [268]. Furthermore, evidence from experimental and epidemiological studies suggests that long-term exposure to low doses of chlorpyrifos may be associated with adverse effects even if the below established no observed adverse effect levels (NOAELs); such subthreshold impacts should be critically considered in future regulatory evaluations [269,270].
(2) Biomonitoring and detection: Human biomonitoring of chlorpyrifos exposure primarily relies on the measurement of urinary 3,5,6-trichloro-2-pyridinol (TCP), the major metabolite of chlorpyrifos. Urinary TCP concentrations correlate strongly with occupational exposure levels, particularly in agricultural and industrial settings, confirming its utility as a reliable exposure biomarker [271]. Urine is the preferred matrix for population-level biomonitoring due to its non-invasive collection and substantially higher TCP concentrations compared to blood or serum. However, it should be noted that TCP can also be present in the environment as a degradation product, which may lead to an overestimation of actual chlorpyrifos intake. While chlorpyrifos and its active metabolite, chlorpyrifos-oxon, can be detected in blood and serum to provide complementary information on the absorbed dose and cholinesterase inhibition potential, these matrices are less practical for large-scale biomonitoring studies [272]. However, TCP is not a fully specific biomarker of chlorpyrifos exposure, as it may also originate from the environmental degradation of chlorpyrifos or from exposure to structurally related organophosphate compounds, which may lead to exposure misclassification [272,273]. Furthermore, due to its relatively short biological half-life of approximately 1–2 days, TCP reflects only recent exposure, limiting its suitability for assessing chronic or cumulative exposure patterns [273]. Advances in analytical techniques, particularly liquid chromatography coupled with tandem mass spectrometry (LC–MS/MS), have significantly improved the sensitivity and detection limits for TCP measurement in biological matrices [274]. A review of 23 human biomonitoring studies across 12 European countries confirmed widespread TCP detection, with median urinary concentrations ranging from 0.06 µg/L among children in Slovenia to 6.72 µg/L among children in Cyprus, highlighting marked geographical variation in chlorpyrifos exposure across the EU [275]. Notably, TCP was detectable in nearly all study populations examined, including pregnant women and children, indicating broad exposure across vulnerable groups [275]. Despite these analytical improvements, interpretation of biomonitoring data remains challenging due to variability in exposure routes, urine sampling strategies, and methods of urine dilution adjustment (e.g., creatinine correction or specific gravity normalization), as well as background environmental levels of TCP. These factors collectively hinder differentiation between acute and chronic exposure and limit comparability across studies.
(3) Sustainable alternatives: Following the EU-wide ban on chlorpyrifos in 2020, research has increasingly focused on biopesticides as viable alternatives to synthetic organophosphorus insecticides. Biopesticides—derived from natural sources including microorganisms, plants, and their metabolites—represent a particularly promising category of alternatives, offering target specificity, environmental sustainability, and an absence of persistent residues, in contrast to conventional synthetic pesticides [276]. Among microbial biopesticides, entomopathogenic fungi and bacteria have received the most attention as potential chlorpyrifos replacements. Specific candidates identified as alternatives to chlorpyrifos include *Beauveria bassiana* [277], *Chromobacterium subtsugae* [278], and azadirachtin-based formulations [279], applicable across a range of crops and target pest species. Studies evaluating *Beauveria bassiana* and *Bacillus thuringiensis* against key agricultural pests such as *Helicoverpa armigera* have demonstrated larval mortality rates of 84–91% and 67–93%, respectively, under laboratory and field conditions [280]. Botanical biopesticides, particularly azadirachtin derived from the neem tree (*Azadirachta indica*), represent another well-documented category of biological alternatives. Azadirachtin exhibits a different mode of action compared to chlorpyrifos. Instead of inducing rapid neurotoxicity, it primarily functions as a growth regulator and feeding deterrent, significantly reducing larval development and delaying the life cycle of target pests [281]. It also interferes with insect feeding behavior and reproduction [282].
  In addition, azadirachtin is characterized by relatively low mammalian toxicity and rapid environmental degradation, resulting in minimal persistence compared with conventional organophosphate insecticides [283]. However, its insecticidal efficacy is often slower and more variable than that of chlorpyrifos. Despite these promising properties, biopesticides are typically most effective when integrated into broader pest management strategies. Incorporating biopesticides into Integrated Pest Management (IPM) programs provides a more holistic approach to maximizing crop yields while reducing reliance on synthetic pesticides and protecting agroecosystems. However, certain characteristics of biopesticides, such as high target specificity, shorter shelf life, and reduced environmental persistence, may limit their applicability, particularly where broad-spectrum pest control is required. Therefore, further research on formulation stability, field efficacy under diverse environmental conditions, and cost-effectiveness is needed before biopesticides can fully replace conventional pesticides in all agricultural applications.
(4) Public awareness and risk communication: Public awareness and risk communication efforts related to chlorpyrifos have been primarily driven by regulatory and public health institutions. In the European Union, the European Food Safety Authority assessment (2019–2020) played a central role by highlighting neurodevelopmental risks and concluding that no safe exposure threshold could be established, which directly contributed to the EU-wide ban implemented under Regulations 2020/17 and 2020/18 [55,284]. This decision was accompanied by targeted communication from the European Commission, aimed at informing agricultural stakeholders and the agrochemical industry about the withdrawal of chlorpyrifos and the need to protect vulnerable populations, particularly children [285]. In May 2025, parties to the Stockholm Convention agreed to list chlorpyrifos as a persistent organic pollutant (POP), marking a shift toward global regulation, with the EU having previously supported stricter controls [10].

Large-scale initiatives such as HBM4EU have increased public awareness by demonstrating the widespread presence of chlorpyrifos metabolites (e.g., TCP) in human urine across different population groups, reinforcing the evidence that exposure persists despite regional restrictions [275]. Additional communication efforts include occupational safety campaigns promoting proper pesticide handling and the use of personal protective equipment, supported by guidelines from organizations such as the World Health Organization and the Food and Agriculture Organization [286]. At the global level, regulatory actions, including the phase-out of chlorpyrifos by Health Canada, the 2021 ban on food crop applications by the United States Environmental Protection Agency (US EPA), and prohibitions in countries such as the United Kingdom (2016), Thailand (2020), and New Zealand (2025), have been accompanied by public communication emphasizing environmental and developmental health risks, particularly for children and pregnant women [287,288]. Collectively, over 40 countries have now banned or severely restricted chlorpyrifos use [289].

These global discrepancies highlight the need for coordinated international communication strategies capable of effectively reaching agricultural workers, policymakers, and the public in countries where chlorpyrifos remains legally in use, and of raising awareness of its well-documented health and environmental risks.

## Figures and Tables

**Table 1:** Physicochemical and environmental fate properties of chlorpyrifos and the metabolite of Chlorpyrifos-oxon (U.S. Environmental Protection Agency).

	Chlorpyrifos	Chlorpyrifos-Oxon
IUPAC Name	O,O-diethyl O-(3,5,6-trichloro-2-pyridyl) phosphorothioate	O,O-diethyl O-3,5,6-trichloropyridin-2-yl phosphate
Chemical Formula	C_9_H_11_Cl_3_NO_3_PS	C_9_H_11_Cl_3_NO_4_P
CAS number	2921-88-2	5598-15-2
Molecular Mass (g/mol)	350.57	334.52
Henry’s Law Constant (atm·m^3^/mol)	6.2 × 10^−6^	5.5 × 10^−9^
Water solubility (20 °C) (ppm)	1.4 mg/mL	26.0 mg/mL
Octanol–water partition coefficient (Log Kow)	4.7	2.89
Hydrolysis half-life (days)	pH 5 (25 °C): 73 pH 7 (25 °C): 72–81 pH 9 (25 °C): 16	pH 4 (20 °C): 37.7 pH 7 (20 °C): 4.8 pH 9 (20 °C): 1.5
Air photolysis half-life (hours) Indirect •OH radical conc. of 1.5 × 10^6^ molecules cm^−3^ Direct [38]	Indirect: 2 Direct: 6	Indirect: 11 Direct: 6
Aerobic soil metabolism half-life range (days) at 25 °C	19–297	<1
Anaerobic soil metabolism half-life range (days) at 25 °C	78–171	No data

**Table 2:** Representative chlorpyrifos exposure levels depending on population, species, and route of exposure.

Target/Matrix	Route of Exposure	Estimated Exposure Concentration/Dose	Issue	References
Humans (general population)	Dietary	<0.001–0.1 µg/kg bw/day	Conclusion on the absence of a safe ADI due to developmental neurotoxicity	[55]
Agricultural workers	Dermal and inhalation	10–100 µg/kg bw/day	Occupational risk assessment; urinary TCP biomonitoring data	[8]
Children (residential)	Non-dietary (dust/surfaces)	4.0–208,000 µg/kg/day	Environmental exposure assessment in residential settings	[59,60,61]
Outdoor Air (USA)	Inhalation	9.2 to 199 ng/m^3^ (0.0037–0.0796 µg/kg/day)	Assessment of CP exposure in a rural agricultural community	[62]
Indoor Air (Colombia)	Inhalation	50–400 ng/m^3^ (0.02–0.16 μg/kg/day)	Determination of CP concentration in indoor air samples	[31]
Prenatal (Colombia)	PBPK modeled dose (maternal/cord)	0.15 µg/kg/day	Comparison of PBPK predictions with maternal and cord blood biomonitoring data	[63]
Aquatic organisms (fish)	Environmental (surface water)	0.5–5.0 µg/L	Ecological risk assessment in agricultural watersheds	[64]
Experimental animals (rat)	Oral (laboratory)	0.001–0.005 mg/kg bw/day	Determination of the no-observed-adverse-effect level	[65]

Abbreviations: ADI, acceptable daily intake; bw, body weight; CP, chlorpyrifos; PBPK, physiologically based pharmacokinetic; TCP, 3,5,6-trichloro-2-pyridinol; USA, United States of America.

## Data Availability

No new data were created or analyzed in this study. Data sharing is not applicable to this article.

## Abbreviations

The following abbreviations are used in this manuscript:

5-HT	Serotonin (5-hydroxytryptamine)
8-OHdG	8-hydroxy-2′-deoxyguanosine
ACh	Acetylcholine
AChE	Acetylcholinesterase
AD	Alzheimer’s disease
Ag3PO4	Silver orthophosphate
AHR	Airway hyperreactivity
ARfD	Acute reference dose
ASD	Autism spectrum disorders
CTD	Characteristic travel distance
BCF	Bioconcentration factor
BDNF	Brain-derived neurotrophic factor
BMD	Bone mineral density
CP	Chlorpyrifos
CPO	Chlorpyrifos-oxon
DETP	Diethyl thiophosphate
DFP	Diisopropyl fluorophosphate
DNT	Developmental neurotoxicity
EDL	Extensor digitorum longus
EFSA	European Food Safety Authority
EPA	Environmental Protection Agency
ERα	Estrogen receptor α
FAAH	Fatty acid amide hydrolase
FGF	Fibroblast growth factor
GWI	Gulf War Illness
HDAC4	Histone deacetylase 4
hNPCs	Human neural progenitor cells
Koa	Octanol–air partition coefficient
Kow	Octanol–water partition coefficient
LH	Luteinizing hormone
LPS	Lipopolysaccharide
MAGL	Monoacylglycerol lipase
MDA	Malondialdehyde
MHC	Myosin heavy chain
ncRNAs	Non-coding RNAs
NGF	Nerve growth factor
NO	Nitric oxide
NO_3_	Nitrate radicals
O_3_	Ozone
OPs	Organophosphates
PD	Parkinson’s disease
POP	Persistent organic pollutant
POPRC	Persistent Organic Pollutants Review Committee
PPARγ	Peroxisome proliferator-activated receptor gamma
ROS	Reactive oxygen species
SMI	Structural model index
TAC	Total antioxidant capacity
Tb.N	Trabecular number
Tb.Sp	Trabecular separation
TOC	Total oxidative capacity
TSA	Total sialic acid
WHO	World Health Organization
ZO-1	Zonula occludens-1
·OH	Hydroxyl radicals

## References

[1] Idris S.B., Ambali S.F., Ayo J.O. (2012). Cytotoxicity of Chlorpyrifos and Cypermethrin: The Ameliorative Effects of Antioxidants. Afr. J. Biotechnol..

[2] Saad H., Elfeky S.A., El-Gamel N.E.A., Abo Dena A.S. (2025). Organophosphate Pesticides: A Review on Classification, Synthesis, Toxicity, Remediation and Analysis. RSC Adv..

[3] John E.M., Shaike J.M. (2015). Chlorpyrifos: Pollution and Remediation. Environ. Chem. Lett..

[4] Koshlukova S.E., Reed N.R. (2014). Chlorpyrifos. Encyclopedia of Toxicology.

[5] Mora-Gutiérrez A., Rubio C., Romero-López Á.A., Rubio-Osornio M., Sabuncuoglu S. (2021). Neurotoxic Effects of Insecticides Chlorpyrifos, Carbaryl, Imidacloprid, in Different Animal Species. Neurotoxicity—New Advances.

[6] Ur Rahman H.U., Asghar W., Nazir W., Sandhu M.A., Ahmed A., Khalid N. (2021). A comprehensive review on chlorpyrifos toxicity with special reference to endocrine disruption: Evidence of mechanisms, exposures and mitigation strategies. Sci. Total Environ..

[7] Wołejko E., Łozowicka B., Jabłońska-Trypuć A., Pietruszyńska M., Wydro U. (2022). Chlorpyrifos Occurrence and Toxicological Risk Assessment: A Review. Int. J. Environ. Res. Public Health.

[8] EPA (Environmental Protection Agency) (2024). 40 CFR Part 180 Chlorpyrifos; Tolerance Revocation.

[9] Silva V., Mol H.G.J., Zomer P., Tienstra M., Ritsema C.J., Geissen V. (2019). Pesticide Residues in European Agricultural Soils—A Hidden Reality Unfolded. Sci. Total Environ..

[10] UNEP (2025). Stockholm Convention on Persistent Organic Pollutants Conference of the Parties to the Stockholm Convention on Persistent Organic Pollutants Twelfth Meeting Geneva Decisions Adopted by the Conference of the Parties to the Stockholm Convention on Persistent Organic Pollutants at Its Twelfth Meeting.

[11] Carrasco Cabrera L., Medina Pastor P. (2021). The 2019 European Union Report on Pesticide Residues in Food. EFSA J..

[12] Carrasco Cabrera L., Di Piazza G., Dujardin B., Medina Pastor P. (2023). The 2021 European Union Report on Pesticide Residues in Food. EFSA J..

[13] Tudi M., Li H., Li H., Wang L., Lyu J., Yang L., Tong S., Yu Q.J., Ruan H.D., Atabila A. (2022). Exposure Routes and Health Risks Associated with Pesticide Application. Toxics.

[14] Mew E.J., Padmanathan P., Konradsen F., Eddleston M., Chang S.S., Phillips M.R., Gunnell D. (2017). The Global Burden of Fatal Self-Poisoning with Pesticides 2006-15: Systematic Review. J. Affect. Disord..

[15] WHO (2019). Preventing Suicide by Phasing Out Highly Hazardous Pesticides.

[16] Ritchie H. (2025). Bans on Highly Toxic Pesticides Could Be an Effective Way to Save Lives from Suicide. Our World Data. https://archive.ourworldindata.org/20260417-161325/pesticide-bans-suicide-prevention.html.

[17] Licata C., Liu L., Mole D., Thorp J., Chand R., Chaulagain S. (2019). Social and Cultural Factors Leading to Suicide Attempt via Organophosphate Poisoning in Nepal. Case Rep. Psychiatry.

[18] Lee W.J., Alavanja M.C.R., Hoppin J.A., Rusiecki J.A., Kamel F., Blair A., Sandler D.P. (2007). Mortality among Pesticide Applicators Exposed to Chlorpyrifos in the Agricultural Health Study. Environ. Health Perspect..

[19] Wu Y.J., Chang S.S., Chen H.Y., Tsai K.F., Lee W.C., Wang I.K., Lee C.H., Chen C.Y., Liu S.H., Weng C.H. (2023). Human Poisoning with Chlorpyrifos and Cypermethrin Pesticide Mixture: Assessment of Clinical Outcome of Cases Admitted in a Tertiary Care Hospital in Taiwan. Int. J. Gen. Med..

[20] Liu H.F., Ku C.H., Chang S.S., Chang C.M., Wang I.K., Yang H.Y., Weng C.H., Huang W.H., Hsu C.W., Yen T.H. (2020). Outcome of Patients with Chlorpyrifos Intoxication. Hum. Exp. Toxicol..

[21] Lincy Juliet K., Adole P.S., Pandit V.R., Vinod K.V. (2018). Serum Paraoxonase 1 Activity in Patients with Organophosphate Poisoning: A Potential Indicator of Prognosis. Asia Pac. J. Med. Toxicol..

[22] Shaffo F.C., Grodzki A.C., Schelegle E.S., Lein P.J. (2018). The Organophosphorus Pesticide Chlorpyrifos Induces Sex-Specific Airway Hyperreactivity in Adult Rats. Toxicol. Sci..

[23] Li B., Eyer P., Eddleston M., Jiang W., Schopfer L.M., Lockridge O. (2013). Protein Tyrosine Adduct in Humans Self-Poisoned by Chlorpyrifos. Toxicol. Appl. Pharmacol..

[24] Sarailoo M., Afshari S., Asghariazar V., Safarzadeh E., Dadkhah M. (2022). Cognitive Impairment and Neurodegenerative Diseases Development Associated with Organophosphate Pesticides Exposure: A Review Study. Neurotox. Res..

[25] Lizé M., Monfort C., Rouget F., Limon G., Durand G., Tillaut H., Chevrier C. (2022). Prenatal exposure to organophosphate pesticides and autism spectrum disorders in 11-year-old children in the French PELAGIE cohort. Environ. Res..

[26] López-Merino E., Cuartero M.I., Esteban J.A., Briz V. (2023). Perinatal Exposure to Pesticides Alters Synaptic Plasticity Signaling and Induces Behavioral Deficits Associated with Neurodevelopmental Disorders. Cell Biol. Toxicol..

[27] Ali S.N., Baqar M., Mumtaz M., Ashraf U., Anwar M.N., Qadir A., Ahmad S.R., Nizami A.S., Jun H. (2020). Organochlorine Pesticides in the Surrounding Soils of POPs Destruction Facility: Source Fingerprinting, Human Health, and Ecological Risks Assessment. Environ. Sci. Pollut. Res..

[28] Mahmood I., Imadi S.R., Shazadi K., Gul A., Hakeem K.R. (2016). Effects of Pesticides on Environment. Plant, Soil and Microbes: Volume 1: Implications in Crop Science.

[29] Zhou W., Li M., Achal V. (2025). A Comprehensive Review on Environmental and Human Health Impacts of Chemical Pesticide Usage. Emerg. Contam..

[30] Smegal D.C., Assessor R. (2000). Human Health Risk Assessment Chlorpyrifos.

[31] Eaton D.L., Daroff R.B., Autrup H., Bridges J., Buffler P., Costa L.G., Coyle J., McKhann G., Mobley W.C., Nadel L. (2008). Review of the Toxicology of Chlorpyrifos with an Emphasis on Human Exposure and Neurodevelopment. Crit. Rev. Toxicol..

[32] Racke K.D. (1993). Environmental fate of chlorpyrifos. Reviews of Environmental Contamination and Toxicology.

[33] Christensen K., Harper B., Luukinen B., Buhl K., Stone D. (2009). Chlorpyrifos General Fact Sheet.

[34] Chai L.K., Wong M.H., Hansen H.C.B. (2013). Degradation of Chlorpyrifos in Humid Tropical Soils. J. Environ. Manag..

[35] Gebremariam S.Y., Beutel M.W., Yonge D.R., Flury M., Harsh J.B. (2012). Adsorption and desorption of chlorpyrifos to soils and sediments. Rev. Environ. Contam. Toxicol..

[36] Wright C.G., Leidy R.B., Dupree H.E. (1991). Chlorpyrifos in the Air and Soil of Houses Four Years after Its Application for Termite Control. Bull. Environ. Contam. Toxicol..

[37] Wright C.G., Leidy R.B., Dupree H.E. (1994). Chlorpyrifos in the Air and Soil of Houses Eight Years after Its Application for Termite Control. Bull. Environ. Contam. Toxicol..

[38] Giesy J.P., Solomon K.R. (2014). Ecological Risk Assessment for Chlorpyrifos in Terrestrial and Aquatic Systems in the United States.

[39] Majewski M.S., Foreman W.T., Goolsbys D.A., Nakagaki N. (1998). Airborne Pesticide Residues Along the Mississippi River. Environ. Sci. Technol..

[40] McConnell L.L., Nelson E., Rice C.P., Baker J.E., Johnson W.E., Harman J.A., Bialek K. (1997). Chlorpyrifos in the Air and Surface Water of Chesapeake Bay: Predictions of Atmospheric Deposition Fluxes. Environ. Sci. Technol..

[41] Balmer J.E., Morris A.D., Hung H., Jantunen L., Vorkamp K., Rigét F., Evans M., Houde M., Muir D.C.G. (2019). Levels and Trends of Current-Use Pesticides (CUPs) in the Arctic: An Updated Review, 2010–2018. Emerg. Contam..

[42] Bigot M., Hawker D.W., Cropp R., Muir D.C.G., Jensen B., Bossi R., Bengtson Nash S.M. (2017). Spring Melt and the Redistribution of Organochlorine Pesticides in the Sea-Ice Environment: A Comparative Study Between Arctic and Antarctic Regions. Environ. Sci. Technol..

[43] Zamora C., Kratzer C.R., Majewski M.S., Knifong D.L. (2003). Diazinon and Chlorpyrifos Loads in Precipitation and Urban and Agricultural Storm Runoff During January and February 2001 in the San Joaquin River Basin, California.

[44] Majewski M.S., Foreman W.T., Goolsby D.A. (2000). Pesticides in the Atmosphere of the Mississippi River Valley, Part I Rain. Sci. Total Environ..

[45] Muñoz A., Vera T., Sidebottom H., Mellouki A., Borrás E., Ródenas M., Clemente E., Vázquez M. (2011). Studies on the Atmospheric Degradation of Chlorpyrifos-Methyl. Environ. Sci. Technol..

[46] Kelly B.C., Ikonomou M.G., Blair J.D., Morin A.E., Gobas F.A.P.C. (2007). Food Web-Specific Biomagnification of Persistent Organic Pollutants. Science.

[47] Morris A.D., Muir D.C.G., Solomon K.R., Letcher R.J., McKinney M.A., Fisk A.T., McMeans B.C., Tomy G.T., Teixeira C., Wang X. (2016). Current-Use Pesticides in Seawater and Their Bioaccumulation in Polar Bear–Ringed Seal Food Chains of the Canadian Arctic. Environ. Toxicol. Chem..

[48] Lavin K.S., Hageman K.J., Marx S.K., Dillingham P.W., Kamber B.S. (2012). Using Trace Elements in Particulate Matter to Identify the Sources of Semivolatile Organic Contaminants in Air at an Alpine Site. Environ. Sci. Technol..

[49] Lester Y., Sabach S., Zivan O., Dubowski Y. (2017). Key Environmental Processes Affecting the Fate of the Insecticide Chloropyrifos Applied to Leaves. Chemosphere.

[50] Henninge L.B., Konieczny R.M., Grabic R., Ferencik M., Bergqvist P.A., Lyngstad E., Berger J. (2019). Screening Programme 2019: Suspected PBT Compounds.

[51] Kushwaha A., Singh G., Sharma M. (2020). Colorimetric Sensing of Chlorpyrifos through Negative Feedback Inhibition of the Catalytic Activity of Silver Phosphate Oxygenase Nanozymes. RSC Adv..

[52] Rathod A.L., Garg R.K. (2017). Chlorpyrifos Poisoning and Its Implications in Human Fatal Cases: A Forensic Perspective with Reference to Indian Scenario. J. Forensic Leg. Med..

[53] Timchalk C., Nolan R.J., Mendrala A.L., Dittenber D.A., Brzak K.A., Mattsson J.L. (2002). A Physiologically based pharmacokinetic and pharmacodynamic (PBPK/PD) model for the organophosphate insecticide chlorpyrifos in rats and humans. Toxicol. Sci..

[54] Slotkin T.A. (2004). Cholinergic Systems in Brain Development and Disruption by Neurotoxicants: Nicotine, Environmental Tobacco Smoke, Organophosphates. Toxicol. Appl. Pharmacol..

[55] European Food Safety Authority (EFSA) (2019). Statement on the Available Outcomes of the Human Health Assessment in the Context of the Pesticides Peer Review of the Active Substance Chlorpyrifos-Methyl. EFSA J..

[56] Rauh V., Arunajadai S., Horton M., Perera F., Hoepner L., Barr D.B., Whyatt R. (2011). Seven-Year Neurodevelopmental Scores and Prenatal Exposure to Chlorpyrifos, a Common Agricultural Pesticide. Environ. Health Perspect..

[57] Sapbamrer R., Hongsibsong S. (2019). Effects of Prenatal and Postnatal Exposure to Organophosphate Pesticides on Child Neurodevelopment in Different Age Groups: A Systematic Review. Environ. Sci. Pollut. Res..

[58] Halpern M.D., Denning P.W. (2015). The role of intestinal epithelial barrier function in the development of NEC. Tissue Barriers.

[59] Simcox N.J., Fenske R.A., Wolz S.A., Lee I.C., Kalman D.A. (1995). Pesticides in household dust and soil: Exposure pathways for children of agricultural families. Environ. Health Perspect..

[60] Gurunathan S., Robson M., Freeman N., Buckley B., Roy A., Meyer R., Bukowski J., Lioy P.J. (1998). Accumulation of chlorpyrifos on residential surfaces and toys accessible to children. Environ. Health Perspect..

[61] Aparicio V., Kaseker J., Scheepers P.T.J., Alaoui A., Figueiredo D.M., Mol H., Silva V., Harkes P., Dos Santos D.R., Geissen V. (2025). Pesticide Contamination in Indoor Home Dust: A Pilot Study of Non-Occupational Exposure in Argentina. Environ. Pollut..

[62] Gibbs J.L., Yost M.G., Negrete M., Fenske R.A. (2017). Passive Sampling for Indoor and Outdoor Exposures to Chlorpyrifos, Azinphos-Methyl, and Oxygen Analogs in a Rural Agricultural Community. Environ. Health Perspect..

[63] Lowe E.R., Poet T.S., Rick D.I., Marty S.M., Mattsson J.I., Timchalk C., Bartels M.J. (2009). The Effect of Plasma Lipids on the Pharmacokinetics of Chlorpyrifos and the Impact on Interpretation of Blood Biomonitoring Data. Toxicol. Sci..

[64] Giddings J.M., Williams W.M., Solomon K.R., Giesy J.P. (2014). Risks to Aquatic Organisms from Use of Chlorpyrifos in the United States. Rev. Environ. Contam. Toxicol..

[65] EFSA (2014). Conclusion on the Peer Review of the Pesticide Human Health Risk Assessment of the Active Substance Chlorpyrifos. EFSA J..

[66] Buratti F.M., Volpe M.T., Meneguz A., Vittozzi L., Testai E. (2003). CYP-Specific Bioactivation of Four Organophosphorothioate Pesticides by Human Liver Microsomes. Toxicol. Appl. Pharmacol..

[67] Croom E.L., Wallace A.D., Hodgson E. (2010). Human Variation in CYP-Specific Chlorpyrifos Metabolism. Toxicology.

[68] D’Agostino J., Zhang H., Kenaan C., Hollenberg P.F. (2015). Mechanism-Based Inactivation of Human Cytochrome P450 2B6 by Chlorpyrifos. Chem. Res. Toxicol..

[69] Costa L.G. (2006). Current Issues in Organophosphate Toxicology. Clin. Chim. Acta.

[70] Foxenberg R.J., McGarrigle B.P., Knaak J.B., Kostyniak P.J., Olson J.R. (2007). Human Hepatic Cytochrome P450-Specific Metabolism of Parathion and Chlorpyrifos. Drug Metab. Dispos..

[71] Tang J., Cao Y., Rose R.L., Brimfield A.A., Dai D., Goldstein J.A., Hodgson E. (2001). Metabolism of chlorpyrifos by human cytochrome P450 isoforms and human, mouse, and rat liver microsomes. Drug Metab. Dispos..

[72] Costa L.G., Giordano G., Cole T.B., Marsillach J., Furlong C.E. (2013). Paraoxonase 1 (PON1) as a Genetic Determinant of Susceptibility to Organophosphate Toxicity. Toxicology.

[73] Dardiotis E., Aloizou A.M., Siokas V., Tsouris Z., Rikos D., Marogianni C., Aschner M., Kovatsi L., Bogdanos D.P., Tsatsakis A. (2019). Paraoxonase-1 Genetic Polymorphisms in Organophosphate Metabolism. Toxicology.

[74] Busby-Hjerpe A.L., Campbell J.A., Smith J.N., Lee S., Poet T.S., Barr D.B., Timchalk C. (2010). Comparative Pharmacokinetics of Chlorpyrifos versus Its Major Metabolites Following Oral Administration in the Rat. Toxicology.

[75] Kopjar N., Žunec S., Mendaš G., Micek V., Kašuba V., Mikolić A., Lovaković B.T., Milić M., Pavičić I., Čermak A.M.M. (2018). Evaluation of Chlorpyrifos Toxicity through a 28-Day Study: Cholinesterase Activity, Oxidative Stress Responses, Parent Compound/Metabolite Levels, and Primary DNA Damage in Blood and Brain Tissue of Adult Male Wistar Rats. Chem. Biol. Interact..

[76] Bakke J.E., Fell V.J., Price C.E. (1976). Rat Urinary Metabolites from o, o-Diethyl-0-(3,5,6-Trichloro-2-Pyridyl) Phosphorothioate. J. Environ. Sci. Health Part B.

[77] Tanvir E.M., Afroz R., Chowdhury M.A.Z., Gan S.H., Karim N., Islam M.N., Khalil M.I. (2016). A Model of Chlorpyrifos Distribution and Its Biochemical Effects on the Liver and Kidneys of Rats. Hum. Exp. Toxicol..

[78] Poulin P., Krishnan K. (1995). An Algorithm for Predicting Tissue: Blood Partition Coefficients of Organic Chemicals from n-Octanol: Water Partition Coefficient Data. J. Toxicol. Environ. Health.

[79] Nolan R.J., Rick D.L., Freshour N.L., Saunders J.H. (1984). Chlorpyrifos: Pharmacokinetics in Human Volunteers. Toxicol. Appl. Pharmacol..

[80] Giesy J.P., Solomon K.R., Cutler G.C., Giddings J.M., Mackay D., Moore D.R.J., Purdy J., Williams W.M. (2014). Ecological Risk Assessment of the Uses of the Organophosphorus Insecticide Chlorpyrifos, in the United States. Reviews of Environmental Contamination and Toxicology.

[81] Gonzalez V., Huen K., Venkat S., Pratt K., Xiang P., Harley K.G., Kogut K., Trujillo C.M., Bradman A., Eskenazi B. (2012). Cholinesterase and Paraoxonase (PON1) Enzyme Activities in Mexican American Mothers and Children from an Agricultural Community. J. Expo. Sci. Environ. Epidemiol..

[82] Timchalk C., Kousba A.A., Poet T.S. (2007). An Age-Dependent Physiologically Based Pharmacokinetic/Pharmacodynamic Model for the Organophosphorus Insecticide Chlorpyrifos in the Preweanling Rat. Toxicol. Sci..

[83] Eskenazi B., Kogut K., Huen K., Harley K.G., Bouchard M., Bradman A., Boyd-Barr D., Johnson C., Holland N. (2014). Organophosphate Pesticide Exposure, PON1, and Neurodevelopment in School-Age Children from the CHAMACOS Study. Environ. Res..

[84] Huen K., Harley K., Bradman A., Eskenazi B., Holland N. (2010). Longitudinal Changes in PON1 Enzymatic Activities in Mexican-American Mothers and Children with Different Genotypes and Haplotypes. Toxicol. Appl. Pharmacol..

[85] Picco E.J., Rubio M.R., Díaz David D.C., Rodríguez C., Boggio J.C. (2008). Pharmacokinetics and Pharmacodynamics of Chlorpyrifos in Male and Female Cattle after Topical Administration. Vet. Res. Commun..

[86] Levin E.D., Addy N., Nakajima A., Christopher N.C., Seidler F.J., Slotkin T.A. (2001). Persistent behavioral consequences of neonatal chlorpyrifos exposure in rats. Dev. Brain Res..

[87] Duirk S.E., Collette T.W. (2006). Degradation of Chlorpyrifos in Aqueous Chlorine Solutions: Pathways, Kinetics, and Modeling. Environ. Sci. Technol..

[88] Sirin G.S., Zhang Y. (2014). How Is Acetylcholinesterase Phosphonylated by Soman? An Ab Initio QM/MM Molecular Dynamics Study. J. Phys. Chem. A.

[89] Bomser J., Casida J.E. (2000). Activation of extracellular signal-regulated kinases (ERK 44/42) by chlorpyrifos oxon in Chinese hamster ovary cells. J. Biochem. Mol. Toxicol..

[90] Thany S.H., Tricoire-Leignel H. (2011). Emerging Pharmacological Properties of Cholinergic Synaptic Transmission: Comparison Between Mammalian and Insect Synaptic and Extrasynaptic Nicotinic Receptors. Curr. Neuropharmacol..

[91] Tsai Y.H., Lein P.J. (2021). Mechanisms of organophosphate neurotoxicity. Curr. Opin. Toxicol..

[92] Greer J.B., Magnuson J.T., Hester K., Giroux M., Pope C., Anderson T., Liu J., Dang V., Denslow N.D., Schlenk D. (2019). Effects of Chlorpyrifos on Cholinesterase and Serine Lipase Activities and Lipid Metabolism in Brains of Rainbow Trout (Oncorhynchus Mykiss). Toxicol. Sci..

[93] Gonçalves A.M.M., Rocha C.P., Marques J.C., Gonçalves F.J.M. (2021). Fatty acids as suitable biomarkers to assess pesticide impacts in freshwater biological scales—A review. Ecol. Indic..

[94] Montanarí C., Franco-Campos F., Taroncher M., Rodríguez-Carrasco Y., Zingales V., Ruiz M.J. (2024). Chlorpyrifos Induces Cytotoxicity via Oxidative Stress and Mitochondrial Dysfunction in HepG2 Cells. Food Chem. Toxicol..

[95] Lu Y.C., Chiang C.Y., Chen S.P., Hsu Y.W., Chen W.Y., Chen C.J., Kuan Y.H., Wu S.W. (2024). Chlorpyrifos-Induced Suppression of the Antioxidative Defense System Leads to Cytotoxicity and Genotoxicity in Macrophages. Environ. Toxicol. Pharmacol..

[96] Parny M., Coste A., Aubouy A., Rahabi M., Prat M., Pipy B., Treilhou M. (2022). Differential immunomodulatory effects of six pesticides of different chemical classes on human monocyte-derived macrophage functions. Food Chem. Toxicol..

[97] Verma R.S., Mehta A., Srivastava N. (2007). In Vivo Chlorpyrifos Induced Oxidative Stress: Attenuation by Antioxidant Vitamins. Pestic. Biochem. Physiol..

[98] Baş H., Kalender Y. (2011). Chlorpyrifos Induced Cardiotoxicity in Rats and the Protective Role of Quercetin and Catechin. Gazi Univ. J. Sci..

[99] Abduh M.S., Alruhaimi R.S., Alqhtani H.A., Hussein O.E., Abukhalil M.H., Kamel E.M., Mahmoud A.M. (2023). Rosmarinic Acid Mitigates Chlorpyrifos-Induced Oxidative Stress, Inflammation, and Kidney Injury in Rats by Modulating SIRT1 and Nrf2/HO-1 Signaling. Life Sci..

[100] Lee J.E., Park J.H., Shin I.C., Koh H.C. (2012). Reactive Oxygen Species Regulated Mitochondria-Mediated Apoptosis in PC12 Cells Exposed to Chlorpyrifos. Toxicol. Appl. Pharmacol..

[101] Alruhaimi R.S. (2023). Betulinic acid protects against cardiotoxicity of the organophosphorus pesticide chlorpyrifos by suppressing oxidative stress, inflammation, and apoptosis in rats. Environ. Sci. Pollut. Res. Int..

[102] Pei H., Liu S., Zeng J., Liu J., Wu H., Chen W., He Z., Du R. (2022). Ros-mediated mitochondrial oxidative stress is involved in the ameliorating effect of ginsenoside GSLS on chlorpyrifos-induced hepatotoxicity in mice. Aging.

[103] Slotkin T.A., Seidler F.J. (2009). Oxidative and Excitatory Mechanisms of Developmental Neurotoxicity: Transcriptional Profiles for Chlorpyrifos, Diazinon, Dieldrin, and Divalent Nickel in PC12 Cells. Environ. Health Perspect..

[104] Weis G.C.C., Assmann C.E., Mostardeiro V.B., Alves A.d.O., da Rosa J.R., Pillat M.M., de Andrade C.M., Schetinger M.R.C., Morsch V.M.M., da Cruz I.B.M. (2021). Chlorpyrifos Pesticide Promotes Oxidative Stress and Increases Inflammatory States in BV-2 Microglial Cells: A Role in Neuroinflammation. Chemosphere.

[105] Gu B., Chen Y., Xu H., Zhan K., Zhu K., Luo H., Huang Y., Zeng H., Zheng W., Tian K. (2025). Subchronic Chlorpyrifos Exposure Induces Thyroid Follicular Cell Pyroptosis to Exacerbate Thyroid Toxicity by Modulating Nrf2/Keap1/NF-ΚB Pathway in Male Mice. J. Inflamm. Res..

[106] Garcia S.J., Seidler F.J., Crumpton T.L., Slotkin T.A. (2001). Does the developmental neurotoxicity of chlorpyrifos involve glial targets? Macromolecule synthesis, adenylyl cyclase signaling, nuclear transcription factors, and formation of reactive oxygen in C6 glioma cells. Brain Res..

[107] Crumpton T.L., Seidler F.J., Slotkin T.A. (2000). Developmental neurotoxicity of chlorpyrifos in vivo and in vitro: Effects on nuclear transcription factors involved in cell replication and differentiation. Brain Res..

[108] Singh N., Lawana V., Luo J., Phong P., Abdalla A., Palanisamy B., Rokad D., Sarkar S., Jin H., Anantharam V. (2018). Organophosphate Pesticide Chlorpyrifos Impairs STAT1 Signaling to Induce Dopaminergic Neurotoxicity: Implications for Mitochondria Mediated Oxidative Stress Signaling Events. Neurobiol. Dis..

[109] Meyer A., Seidler F.J., Slotkin T.A. (2004). Developmental Effects of Chlorpyrifos Extend beyond Neurotoxicity: Critical Periods for Immediate and Delayed-Onset Effects on Cardiac and Hepatic Cell Signaling. Environ. Health Perspect..

[110] Yin X.H., Zhu G.N., Li X.B., Liu S.Y. (2009). Genotoxicity Evaluation of Chlorpyrifos to Amphibian Chinese Toad (Amphibian: Anura) by Comet Assay and Micronucleus Test. Mutat. Res. Genet. Toxicol. Environ. Mutagen..

[111] Ali D., Nagpure N.S., Kumar S., Kumar R., Kushwaha B., Lakra W.S. (2009). Assessment of Genotoxic and Mutagenic Effects of Chlorpyrifos in Freshwater Fish *Channa punctatus* (Bloch) Using Micronucleus Assay and Alkaline Single-Cell Gel Electrophoresis. Food Chem. Toxicol..

[112] Ismail M., Khan Q.M., Ali R., Ali T., Mobeen A. (2014). Genotoxicity of Chlorpyrifos in Freshwater Fish *Labeo rohita* Using Alkaline Single-Cell Gel Electrophoresis (Comet) Assay. Drug Chem. Toxicol..

[113] Ali D., Nagpure N.S., Kumar S., Kumar R., Kushwaha B. (2008). Genotoxicity Assessment of Acute Exposure of Chlorpyrifos to Freshwater Fish *Channa punctatus* (Bloch) Using Micronucleus Assay and Alkaline Single-Cell Gel Electrophoresis. Chemosphere.

[114] Li D., Huang Q., Lu M., Zhang L., Yang Z., Zong M., Tao L. (2015). The Organophosphate Insecticide Chlorpyrifos Confers Its Genotoxic Effects by Inducing DNA Damage and Cell Apoptosis. Chemosphere.

[115] Ojha A., Srivastava N. (2014). In Vitro Studies on Organophosphate Pesticides Induced Oxidative DNA Damage in Rat Lymphocytes. Mutat. Res. Genet. Toxicol. Environ. Mutagen..

[116] Kašuba V., Micek V., Milić M., Želježić D., Katić A. (2022). The Effect of Low Doses of Chlorpyrifos on Blood and Bone Marrow Cells in Wistar Rats. Arh. Hig. Rada Toksikol..

[117] Ezzi L., Belhadj Salah I., Haouas Z., Sakly A., Grissa I., Chakroun S., Kerkeni E., Hassine M., Mehdi M., Ben Cheikh H. (2016). Histopathological and Genotoxic Effects of Chlorpyrifos in Rats. Environ. Sci. Pollut. Res..

[118] Khan S., Qayoom I., Balkhi M.H., Abubakr A., Rashid S., Alsaffar R.M., Rehman M.U. (2022). Behavioural Incongruities in Juvenile Cyprinus Carpio Exposed to Organophosphate Compounds. Heliyon.

[119] Mitkovska V., Chassovnikarova T. (2020). Chlorpyrifos Levels within Permitted Limits Induce Nuclear Abnormalities and DNA Damage in the Erythrocytes of the Common Carp. Environ. Sci. Pollut. Res..

[120] Želježić D., Mladinić M., Žunec S., Lucić Vrdoljak A., Kašuba V., Tariba B., Živković T., Marjanović A.M., Pavičić I., Milić M. (2016). Cytotoxic, Genotoxic and Biochemical Markers of Insecticide Toxicity Evaluated in Human Peripheral Blood Lymphocytes and an HepG2 Cell Line. Food Chem. Toxicol..

[121] Sandhu M.A., Saeed A.A., Khilji M.S., Ahmed A., Latif M.S., Khalid N. (2013). Genotoxicity evaluation of chlorpyrifos: A gender related approach in regular toxicity testing. J. Toxicol. Sci..

[122] Imam A., Sulaiman N.A., Oyewole A.L., Chengetanai S., Williams V., Ajibola M.I., Folarin R.O., Muhammad A.S., Shittu S.T.T., Ajao M.S. (2018). Chlorpyrifos- and Dichlorvos-Induced Oxidative and Neurogenic Damage Elicits Neuro-Cognitive Deficits and Increases Anxiety-like Behavior in Wild-Type Rats. Toxics.

[123] Imam A., Sulaiman N., Oyewole A., Amin A., Shittu S., Ajao M. (2018). Pro-Neurogenic and Antioxidant Efficacy of *Nigella sativa* Oil Reduced Vulnerability to Cholinesterase Dysfunction and Disruption in Amygdala-Dependent Behaviours in Chlorpyrifos Exposure. J. Krishna Inst. Med. Sci. Univ..

[124] Day J.J., Sweatt J.D. (2011). Epigenetic modifications in neurons are essential for formation and storage of behavioral memory. Neuropsychopharmacology.

[125] Kim H.Y., Wegner S.H., Van Ness K.P., Park J.J., Pacheco S.E., Workman T., Hong S., Griffith W., Faustman E.M. (2016). Differential Epigenetic Effects of Chlorpyrifos and Arsenic in Proliferating and Differentiating Human Neural Progenitor Cells. Reprod. Toxicol..

[126] Chiu K.C., Sisca F., Ying J.H., Tsai W.J., Hsieh W.S., Chen P.C., Liu C.Y. (2021). Prenatal Chlorpyrifos Exposure in Association with PPARγ H3K4me3 and DNA Methylation Levels and Child Development. Environ. Pollut..

[127] Mansukhani M., Ganguli N., Majumdar S.S., Sharma S.S. (2026). Chronic Oral Exposure to Chlorpyrifos Disrupts Hepatic Epigenetic Regulation and Induces Metabolic Dysfunction in Mice Author Links Open Overlay PanelMeenakshi Mansukhani. Toxicology.

[128] Konopka W., Kiryk A., Novak M., Herwerth M., Parkitna J.R., Wawrzyniak M., Kowarsch A., Michaluk P., Dzwonek J., Arnsperger T. (2010). MicroRNA Loss Enhances Learning and Memory in Mice. J. Neurosci..

[129] El Fatimy R., Boulaassafre S., Bouchmaa N., El Khayari A., Vergely C., Malka G., Rochette L. (2021). The emerging role of miRNA-132/212 cluster in neurologic and cardiovascular diseases: Neuroprotective role in cells with prolonged longevity. Mech. Ageing Dev..

[130] Wanet A., Tacheny A., Arnould T., Renard P. (2012). MiR-212/132 Expression and Functions: Within and beyond the Neuronal Compartment. Nucleic Acids Res..

[131] Lee Y.S., Lewis J.A., Ippolito D.L., Hussainzada N., Lein P.J., Jackson D.A., Stallings J.D. (2016). Repeated Exposure to Neurotoxic Levels of Chlorpyrifos Alters Hippocampal Expression of Neurotrophins and Neuropeptides. Toxicology.

[132] Miguel V., Cui J.Y., Daimiel L., Espinosa-Díez C., Fernández-Hernando C., Kavanagh T.J., Lamas S. (2018). The Role of MicroRNAs in Environmental Risk Factors, Noise-Induced Hearing Loss, and Mental Stress. Antioxid. Redox Signal..

[133] Costa C., Teodoro M., Rugolo C.A., Alibrando C., Giambò F., Briguglio G., Fenga C. (2020). MicroRNAs Alteration as Early Biomarkers for Cancer and Neurodegenerative Diseases: New Challenges in Pesticides Exposure. Toxicol. Rep..

[134] Miao Z., Miao Z., Teng X., Xu S. (2022). Chlorpyrifos Triggers Epithelioma Papulosum Cyprini Cell Pyroptosis via MiR-124-3p/CAPN1 Axis. J. Hazard. Mater..

[135] Li W., Jiang Y., Wang Y., Yang S., Bi X., Pan X., Ma A., Li W. (2018). MiR-181b Regulates Autophagy in a Model of Parkinson’s Disease by Targeting the PTEN/Akt/MTOR Signaling Pathway. Neurosci. Lett..

[136] Kim T., Valera E., Desplats P. (2019). Alterations in Striatal MicroRNA-MRNA Networks Contribute to Neuroinflammation in Multiple System Atrophy. Mol. Neurobiol..

[137] Indrieri A., Carrella S., Carotenuto P., Banfi S., Franco B. (2020). The Pervasive Role of the miR-181 Family in Development, Neurodegeneration, and Cancer. Int. J. Mol. Sci..

[138] Wu Q., Yuan X., Bai J., Han R., Li Z., Zhang H., Xiu R. (2019). MicroRNA-181a protects against pericyte apoptosis via directly targeting FOXO1: Implication for ameliorated cognitive deficits in APP/PS1 mice. Aging.

[139] Zhao M.W., Yang P., Zhao L.L. (2019). Chlorpyrifos Activates Cell Pyroptosis and Increases Susceptibility on Oxidative Stress-Induced Toxicity by MiR-181/SIRT1/PGC-1α/Nrf2 Signaling Pathway in Human Neuroblastoma SH-SY5Y Cells: Implication for Association Between Chlorpyrifos and Parkinson’s Disease. Environ. Toxicol..

[140] Tang J., Carr R.L., Chambers J.E. (1999). Changes in rat brain cholinesterase activity and muscarinic receptor density during and after repeated oral exposure to chlorpyrifos in early postnatal development. Toxicol. Sci..

[141] Abdollahi M., Mostafalou S., Pournourmohammadi S., Shadnia S. (2004). Oxidative Stress and Cholinesterase Inhibition in Saliva and Plasma of Rats Following Subchronic Exposure to Malathion. Comp. Biochem. Physiol.-C Toxicol. Pharmacol..

[142] Teleanu D.M., Niculescu A.G., Lungu I.I., Radu C.I., Vladâcenco O., Roza E., Costăchescu B., Grumezescu A.M., Teleanu R.I. (2022). An Overview of Oxidative Stress, Neuroinflammation, and Neurodegenerative Diseases. Int. J. Mol. Sci..

[143] Cobley J.N., Fiorello M.L., Bailey D.M. (2018). 13 reasons why the brain is susceptible to oxidative stress. Redox Biol..

[144] Lorke D.E., Oz M. (2025). A Review on Oxidative Stress in Organophosphate-Induced Neurotoxicity. Int. J. Biochem. Cell Biol..

[145] Abolaji A.O., Ojo M., Afolabi T.T., Arowoogun M.D., Nwawolor D., Farombi E.O. (2017). Protective Properties of 6-Gingerol-Rich Fraction from *Zingiber officinale* (Ginger) on Chlorpyrifos-Induced Oxidative Damage and Inflammation in the Brain, Ovary and Uterus of Rats. Chem. Biol. Interact..

[146] Houldsworth A. (2024). Role of oxidative stress in neurodegenerative disorders: A review of reactive oxygen species and prevention by antioxidants. Brain Commun..

[147] Olufunmilayo E.O., Gerke-Duncan M.B., Holsinger R.M.D. (2023). Oxidative Stress and Antioxidants in Neurodegenerative Disorders. Antioxidants.

[148] Deveci H.A., Karapehlivan M. (2018). Chlorpyrifos-Induced Parkinsonian Model in Mice: Behavior, Histopathology and Biochemistry. Pestic. Biochem. Physiol..

[149] Middlemore-Risher M.L., Adam B.L., Lambert N.A., Terry A.V. (2011). Effects of Chlorpyrifos and Chlorpyrifos-Oxon on the Dynamics and Movement of Mitochondria in Rat Cortical Neurons. J. Pharmacol. Exp. Ther..

[150] Yamada S., Kubo Y., Yamazaki D., Sekino Y., Kanda Y. (2017). Chlorpyrifos Inhibits Neural Induction via Mfn1-Mediated Mitochondrial Dysfunction in Human Induced Pluripotent Stem Cells. Sci. Rep..

[151] Lin J.W., Fu S.C., Liu J.M., Liu S.H., Lee K.I., Fang K.M., Hsu R.J., Huang C.F., Liu K.M., Chang K.C. (2023). Chlorpyrifos induces neuronal cell death via both oxidative stress and Akt activation downstream-regulated CHOP-triggered apoptotic pathways. Toxicol. Vitr..

[152] Dickey B., Madhu L.N., Shetty A.K. (2021). Gulf War Illness: Mechanisms Underlying Brain Dysfunction and Promising Therapeutic Strategies. Pharmacol. Ther..

[153] Locker A.R., Michalovicz L.T., Kelly K.A., Miller J.V., Miller D.B., O’Callaghan J.P. (2017). Corticosterone Primes the Neuroinflammatory Response to Gulf War Illness-Relevant Organophosphates Independently of Acetylcholinesterase Inhibition. J. Neurochem..

[154] Lesiak A., Zhu M., Chen H., Appleyard S.M., Impey S., Lein P.J., Wayman G.A. (2014). The Environmental Neurotoxicant PCB 95 Promotes Synaptogenesis via Ryanodine Receptor-Dependent MiR132 Upregulation. J. Neurosci..

[155] Wang R., Chen L., Zhang Y., Sun B., Liang M. (2024). Expression Changes of miRNAs in Humans and Animal Models of Amyotrophic Lateral Sclerosis and Their Potential Application for Clinical Diagnosis. Life.

[156] Yu G., Su Q., Chen Y., Wu L., Wu S., Li H. (2021). Epigenetics in neurodegenerative disorders induced by pesticides. Genes Environ..

[157] Shih D.M., Gu L., Xia Y.R., Navab M., Li W.F., Hama S., Castellani L.W., Furlong C.E., Costa L.G., Fogelman A.M. (1998). Mice Lacking Serum Paraoxonase Are Susceptible to Organophosphate Toxicity and Atherosclerosis. Nature.

[158] Cole T.B., Walter B.J., Shih D.M., Tward A.D., Lusis A.J., Timchalk C., Richter R.J., Costa L.G., Furlong C.E. (2005). Toxicity of chlorpyrifos and chlorpyrifos oxon in a transgenic mouse model of the human paraoxonase (PON1) Q192R polymorphism. Pharmacogenet. Genom..

[159] Manthripragada A.D., Costello S., Cockburn M.G., Bronstein J.M., Ritz B. (2010). Paraoxonase 1, Agricultural Organophosphate Exposure, and Parkinson Disease. Epidemiology.

[160] Nielsen S.S., Mueller B.A., De Roos A.J., Viernes H.M.A., Farin F.M., Checkoway H. (2005). Risk of Brain Tumors in Children and Susceptibility to Organophosphorus Insecticides: The Potential Role of Paraoxonase (PON1). Environ. Health Perspect..

[161] Cole T.B., Jampsa R.L., Walter B.J., Arndt T.L., Richter R.J., Shih D.M., Tward A., Lusis A.J., Jack R.M., Costa L.G. (2003). Expression of Human Paraoxonase (PON1) during Development. Pharmacogenetics.

[162] Harley K.G., Huen K., Schall R.A., Holland N.T., Bradman A., Barr D.B., Eskenazi B. (2011). Association of Organophosphate Pesticide Exposure and Paraoxonase with Birth Outcome in Mexican-American Women. PLoS ONE.

[163] Eskenazi B., Huen K., Marks A., Harley K.G., Bradman A., Barr D.B., Holland N. (2010). PON1 and Neurodevelopment in Children from the Chamacos Study Exposed to Organophosphate Pesticides in Utero. Environ. Health Perspect..

[164] Engel S.M., Wetmur J., Chen J., Zhu C., Barr D.B., Canfield R.L., Wolff M.S. (2011). Prenatal Exposure to Organophosphates, Paraoxonase 1, and Cognitive Development in Childhood. Environ. Health Perspect..

[165] Tomás M., Latorre G., Sentí M., Marrugat J. (2004). Función antioxidante de las lipoproteínas de alta densidad: Un nuevo paradigma en la arteriosclerosis [The antioxidant function of high density lipoproteins: A new paradigm in atherosclerosis]. Rev. Esp. Cardiol..

[166] Taler-Verčič A., Goličnik M., Bavec A. (2020). The Structure and Function of Paraoxonase-1 and Its Comparison to Paraoxonase-2 and -3. Molecules.

[167] Khalaf F.K., Connolly J., Khatib-Shahidi B., Albehadili A., Tassavvor I., Ranabothu M., Eid N., Dube P., Khouri S.J., Malhotra D. (2023). Paraoxonases at the Heart of Neurological Disorders. Int. J. Mol. Sci..

[168] Costa L.G., Cole T.B., Vitalone A., Furlong C.E. (2005). Measurement of Paraoxonase (PON1) Status as a Potential Biomarker of Susceptibility to Organophosphate Toxicity. Clin. Chim. Acta.

[169] Costa L.G., Giordano G., Furlong C.E. (2011). Pharmacological and Dietary Modulators of Paraoxonase 1 (PON1) Activity and Expression: The Hunt Goes on. Biochem. Pharmacol..

[170] Belin A.C., Ran C., Anvret A., Paddock S., Westerlund M., Håkansson A., Nissbrandt H., Söderkvist P., Dizdar N., Ahmadi A. (2012). Association of a Protective Paraoxonase 1 (PON1) Polymorphism in Parkinson’s Disease. Neurosci. Lett..

[171] Clarimon J., Eerola J., Hellström O., Tienari P.J., Singleton A. (2004). Paraoxonase 1 (PON1) Gene Polymorphisms and Parkinson’s Disease in a Finnish Population. Neurosci. Lett..

[172] Farag A.T., Radwan A.H., Sorour F., El Okazy A., El-Agamy E.S., El-Sebae A.E.K. (2010). Chlorpyrifos Induced Reproductive Toxicity in Male Mice. Reprod. Toxicol..

[173] Rauh V.A., Garfinkel R., Perera F.P., Andrews H.F., Hoepner L., Barr D.B., Whitehead R., Tang D., Whyatt R.W. (2006). Impact of Prenatal Chlorpyrifos Exposure on Neurodevelopment in the First 3 Years of Life among Inner-City Children. Pediatrics.

[174] Burke R.D., Todd S.W., Lumsden E., Mullins R.J., Mamczarz J., Fawcett W.P., Gullapalli R.P., Randall W.R., Pereira E.F.R., Albuquerque E.X. (2017). Developmental Neurotoxicity of the Organophosphorus Insecticide Chlorpyrifos: From Clinical Findings to Preclinical Models and Potential Mechanisms. J. Neurochem..

[175] Posta E., Fekete I., Varkonyi I., Zold E., Barta Z. (2024). The Versatile Role of Peroxisome Proliferator-Activated Receptors in Immune-Mediated Intestinal Diseases. Cells.

[176] Villapol S. (2018). Roles of Peroxisome Proliferator-Activated Receptor Gamma on Brain and Peripheral Inflammation. Cell. Mol. Neurobiol..

[177] Peterson B.S., Delavari S., Bansal R., Sawardekar S., Gupte C., Andrews H., Hoepner L.A., Garcia W., Perera F., Rauh V. (2025). Brain Abnormalities in Children Exposed Prenatally to the Pesticide Chlorpyrifos. JAMA Neurol..

[178] Lee J.E., Park J.H., Jang S.J., Koh H.C. (2014). Rosiglitazone Inhibits Chlorpyrifos-Induced Apoptosis via Modulation of the Oxidative Stress and Inflammatory Response in SH-SY5Y Cells. Toxicol. Appl. Pharmacol..

[179] Slotkin T.A., Seidler F.J., Fumagalli F. (2007). Exposure to Organophosphates Reduces the Expression of Neurotrophic Factors in Neonatal Rat Brain Regions: Similarities and Differences in the Effects of Chlorpyrifos and Diazinon on the Fibroblast Growth Factor Superfamily. Environ. Health Perspect..

[180] Otto D., Unsicker K. (1990). Basic FGF reverses chemical and morphological deficits in the nigrostriatal system of MPTP-treated mice. J. Neurosci..

[181] Shults C.W., Ray J., Tsuboi K., Gage F.H. (2000). Fibroblast growth factor-2-producing fibroblasts protect the nigrostriatal dopaminergic system from 6-hydroxydopamine. Brain Res..

[182] Tooyama I., Kawamata T., Walker D., Yamada T., Hanai K., Kimura H., Iwane M., Igarashi K., McGeer E.G., McGeer P.L. (1993). Loss of basic fibroblast growth factor in substantia nigra neurons in Parkinson’s disease. Neurology.

[183] Slotkin T.A., Seidler F.J., Ryde I.T., Yanai J. (2008). Developmental Neurotoxic Effects of Chlorpyrifos on Acetylcholine and Serotonin Pathways in an Avian Model. Neurotoxicol. Teratol..

[184] Flaskos J. (2012). The Developmental Neurotoxicity of Organophosphorus Insecticides: A Direct Role for the Oxon Metabolites. Toxicol. Lett..

[185] Howard A.S., Bucelli R., Jett D.A., Bruun D., Yang D., Lein P.J. (2005). Chlorpyrifos Exerts Opposing Effects on Axonal and Dendritic Growth in Primary Neuronal Cultures. Toxicol. Appl. Pharmacol..

[186] Jiang W., Duysen E.G., Hansen H., Shlyakhtenko L., Schopfer L.M., Lockridge O. (2010). Mice Treated with Chlorpyrifos or Chlorpyrifos Oxon Have Organophosphorylated Tubulin in the Brain and Disrupted Microtubule Structures, Suggesting a Role for Tubulin in Neurotoxicity Associated with Exposure to Organophosphorus Agents. Toxicol. Sci..

[187] Schopfer L.M., Lockridge O. (2018). Chlorpyrifos Oxon Promotes Tubulin Aggregation via Isopeptide Cross-Linking Between Diethoxyphospho-Lys and Glu or ASP: Implications for Neurotoxicity. J. Biol. Chem..

[188] Flaskos J., Nikolaidis E., Harris W., Sachana M., Hargreaves A.J. (2011). Effects of Sub-Lethal Neurite Outgrowth Inhibitory Concentrations of Chlorpyrifos Oxon on Cytoskeletal Proteins and Acetylcholinesterase in Differentiating N2a Cells. Toxicol. Appl. Pharmacol..

[189] Huff R.A., Abou-Donia M.B. (1995). In Vitro effect of chlorpyrifos oxon on muscarinic receptors and adenylate cyclase. Neurotoxicology.

[190] Olivier K., Liu J., Pope C. (2001). Inhibition of forskolin-stimulated cAMP formation in vitro by paraoxon and chlorpyrifos oxon in cortical slices from neonatal, juvenile, and adult rats. J. Biochem. Mol. Toxicol..

[191] Katz E.J., Cortes V.I., Eldefrawi M.E., Eldefrawi A.T. (1997). Chlorpyrifos, parathion, and their oxons bind to and desensitize a nicotinic acetylcholine receptor: Relevance to their toxicities. Toxicol. Appl. Pharmacol..

[192] Quistad G.B., Nomura D.K., Sparks S.E., Segall Y., Casida J.E. (2002). Cannabinoid CB1 receptor as a target for chlorpyrifos oxon and other organophosphorus pesticides. Toxicol. Lett..

[193] Howard M.D., Mirajkar N., Karanth S., Pope C.N. (2007). Comparative Effects of Oral Chlorpyrifos Exposure on Cholinesterase Activity and Muscarinic Receptor Binding in Neonatal and Adult Rat Heart. Toxicology.

[194] Bomser J.A., Casida J.E. (2001). Diethylphosphorylation of Rat Cardiac M2 Muscarinic Receptor by Chlorpyrifos Oxon in vitro. Toxicol. Lett..

[195] Casida J.E., Quistad G.B. (2004). Organophosphate Toxicology: Safety Aspects of Nonacetylcholinesterase Secondary Targets. Chem. Res. Toxicol..

[196] Carr R.L., Adams A.L., Kepler D.R., Ward A.B., Ross M.K. (2013). Induction of Endocannabinoid Levels in Juvenile Rat Brain Following Developmental Chlorpyrifos Exposure. Toxicol. Sci..

[197] Carr R.L., Borazjani A., Ross M.K. (2011). Effect of Developmental Chlorpyrifos Exposure, on Endocannabinoid Metabolizing Enzymes, in the Brain of Juvenile Rats. Toxicol. Sci..

[198] Sogorb M.A., Vilanova E., Satoh T., Gupta R.C. (2011). Detoxication of Anticholinesterase Pesticides. Anticholinesterase Pesticides: Metabolism, Neurotoxicity, and Epidemiology.

[199] Sogorb M.A., Vilanova E. (2002). Enzymes involved in the detoxification of organophosphorus, carbamate and pyrethroid insecticides through hydrolysis. Toxicol. Lett..

[200] Bomser J.A., Quistad G.B., Casida J.E. (2002). Chlorpyrifos Oxon Potentiates Diacylglycerol-Induced Extracellular Signal-Regulated Kinase (ERK 44/42) Activation, Possibly by Diacylglycerol Lipase Inhibition. Toxicol. Appl. Pharmacol..

[201] Monnet-Tschudi F., Zurich M.G., Schilter B., Costa L.G., Honegger P. (2000). Maturation-Dependent Effects of Chlorpyrifos and Parathion and Their Oxygen Analogs on Acetylcholinesterase and Neuronal and Glial Markers in Aggregating Brain Cell Cultures. Toxicol. Appl. Pharmacol..

[202] Sun J., Nan G. (2017). The Extracellular Signal-Regulated Kinase 1/2 Pathway in Neurological Diseases: A Potential Therapeutic Target (Review). Int. J. Mol. Med..

[203] Mebratu Y., Tesfaigzi Y. (2009). How ERK1/2 activation controls cell proliferation and cell death: Is subcellular localization the answer?. Cell Cycle.

[204] Šulc L., Janoš T., Figueiredo D., Ottenbros I., Šenk P., Mikeš O., Huss A., Čupr P. (2022). Pesticide Exposure among Czech Adults and Children from the CELSPAC-SPECIMEn Cohort: Urinary Biomarker Levels and Associated Health Risks. Environ. Res..

[205] Wang L., Liu Z., Zhang J., Wu Y., Sun H. (2016). Chlorpyrifos Exposure in Farmers and Urban Adults: Metabolic Characteristic, Exposure Estimation, and Potential Effect of Oxidative Damage. Environ. Res..

[206] Janoš T., Ottenbros I., Bláhová L., Šenk P., Šulc L., Pálešová N., Sheardová J., Vlaanderen J., Čupr P. (2023). Effects of Pesticide Exposure on Oxidative Stress and DNA Methylation Urinary Biomarkers in Czech Adults and Children from the CELSPAC-SPECIMEn Cohort. Environ. Res..

[207] Tweedale A.C. (2017). The Inadequacies of Pre-Market Chemical Risk Assessment’s Toxicity Studies—The Implications. J. Appl. Toxicol..

[208] Sheppard L., McGrew S., Fenske R.A. (2020). Flawed Analysis of an Intentional Human Dosing Study and Its Impact on Chlorpyrifos Risk Assessments. Environ. Int..

[209] Mie A., Rudén C., Grandjean P. (2018). Safety of Safety Evaluation of Pesticides: Developmental Neurotoxicity of Chlorpyrifos and Chlorpyrifos-Methyl. Environ. Health.

[210] Ventura C., Nieto M.R.R., Bourguignon N., Lux-Lantos V., Rodriguez H., Cao G., Randi A., Cocca C., Núñez M. (2016). Pesticide Chlorpyrifos Acts as an Endocrine Disruptor in Adult Rats Causing Changes in Mammary Gland and Hormonal Balance. J. Steroid Biochem. Mol. Biol..

[211] Lasagna M., Ventura C., Hielpos M.S., Mardirosian M.N., Martín G., Miret N., Randi A., Núñez M., Cocca C. (2022). Endocrine Disruptor Chlorpyrifos Promotes Migration, Invasion, and Stemness Phenotype in 3D Cultures of Breast Cancer Cells and Induces a Wide Range of Pathways Involved in Cancer Progression. Environ. Res..

[212] De Angelis S., Tassinari R., Maranghi F., Eusepi A., Di Virgilio A., Chiarotti F., Ricceri L., Venerosi Pesciolini A., Gilardi E., Moracci G. (2009). Developmental Exposure to Chlorpyrifos Induces Alterations in Thyroid and Thyroid Hormone Levels without Other Toxicity Signs in CD-1 Mice. Toxicol. Sci..

[213] Jeong S.H., Kim B.Y., Kang H.G., Ku H.O., Cho J.H. (2006). Effect of Chlorpyrifos-Methyl on Steroid and Thyroid Hormones in Rat F0- and F1-Generations. Toxicology.

[214] Viswanath G., Chatterjee S., Dabral S., Nanguneri S.R., Divya G., Roy P. (2010). Anti-Androgenic Endocrine Disrupting Activities of Chlorpyrifos and Piperophos. J. Steroid Biochem. Mol. Biol..

[215] Peluso T., Nittoli V., Reale C., Porreca I., Russo F., Roberto L., Giacco A., Silvestri E., Mallardo M., De Felice M. (2023). Chronic Exposure to Chlorpyrifos Damages Thyroid Activity and Imbalances Hepatic Thyroid Hormones Signaling and Glucose Metabolism: Dependency of T3-FOXO1 Axis by Hyperglycemia. Int. J. Mol. Sci..

[216] Pastorino M., Desiderio A., Perrella E., Campitelli M., Nigro C., Peluso T., De Felice M., Ambrosino C., Beguinot F., Miele C. (2026). The Endocrine Disruptor Chlorpyrifos Alters Hypothalamic Npy and Agrp Expression via ERβ-Dependent Regulation in vitro and In Vivo. Front. Endocrinol..

[217] Peris-Sampedro F., Cabré M., Basaure P., Reverte I., Domingo J.L., Teresa Colomina M. (2015). Adulthood Dietary Exposure to a Common Pesticide Leads to an Obese-like Phenotype and a Diabetic Profile in ApoE3 Mice. Environ. Res..

[218] Wang B., Tsakiridis E.E., Zhang S., Llanos A., Desjardins E.M., Yabut J.M., Green A.E., Day E.A., Smith B.K., Lally J.S.V. (2021). The Pesticide Chlorpyrifos Promotes Obesity by Inhibiting Diet-Induced Thermogenesis in Brown Adipose Tissue. Nat. Commun..

[219] Bernabò I., Gallo L., Sperone E., Tripepi S., Brunelli E. (2011). Survival, Development, and Gonadal Differentiation in *Rana dalmatina* Chronically Exposed to Chlorpyrifos. J. Exp. Zool. A Ecol. Genet. Physiol..

[220] Canesi L., Negri A., Barmo C., Banni M., Gallo G., Viarengo A., Dondero F. (2011). The Organophosphate Chlorpyrifos Interferes with the Responses to 17β-Estradiol in the Digestive Gland of the Marine Mussel Mytilus Galloprovincialis. PLoS ONE.

[221] Fouyet S., Olivier E., Leproux P., Boutefnouchet S., Dutot M., Rat P. (2022). Cocktail Effect of Endocrine Disrupting Chemicals: Application to Chlorpyrifos in Lavender Essential Oils. Int. J. Environ. Res. Public Health.

[222] Hazarika J., Ganguly M., Borgohain G., Baruah I., Sarma S., Bhuyan P., Mahanta R. (2020). Endocrine Disruption: Molecular Interactions of Chlorpyrifos and Its Degradation Products with Estrogen Receptor. Struct. Chem..

[223] Song X., Li X., Wang Y., Wu Y.J. (2024). Involvement of Gut Microbiota in Chlorpyrifos-Induced Subchronic Toxicity in Mice. Arch. Toxicol..

[224] Deng Y., Zhang Y., Lu Y., Zhao Y., Ren H. (2016). Hepatotoxicity and Nephrotoxicity Induced by the Chlorpyrifos and Chlorpyrifos-Methyl Metabolite, 3,5,6-Trichloro-2-Pyridinol, in Orally Exposed Mice. Sci. Total Environ..

[225] Biedermann L., Rogler G. (2015). The Intestinal Microbiota: Its Role in Health and Disease. Eur. J. Pediatr..

[226] Zhang Y., Jia Q., Hu C., Han M., Guo Q., Li S., Bo C., Zhang Y., Qi X., Sai L. (2021). Effects of Chlorpyrifos Exposure on Liver Inflammation and Intestinal Flora Structure in Mice. Toxicol. Res..

[227] Zhao Y., Zhang Y., Wang G., Han R., Xie X. (2016). Effects of Chlorpyrifos on the Gut Microbiome and Urine Metabolome in Mouse (*Mus musculus*). Chemosphere.

[228] Walker A.W., Sanderson J.D., Churcher C., Parkes G.C., Hudspith B.N., Rayment N., Brostoff J., Parkhill J., Dougan G., Petrovska L. (2011). High-Throughput Clone Library Analysis of the Mucosa-Associated Microbiota Reveals Dysbiosis and Differences Between Inflamed and Non-Inflamed Regions of the Intestine in Inflammatory Bowel Disease. BMC Microbiol..

[229] Nichols R.G., Rimal B., Hao F., Peters J.M., Davenport E.R., Patterson A.D. (2024). Chlorpyrifos Modulates the Mouse Gut Microbiota and Metabolic Activity. Environ. Int..

[230] Durairaj K., Gajendran B., Manivel G., Gnanam H., Vasudevan S.A., Seenivasan S.N., Pandian S., Shanmugarajan S., Vily-Petit J., Mariappan K.T. (2025). Exposure to chlorpyrifos pesticide at a realistic dose modulates gut microbiome and induces non-obese associated diabetes. Environ. Sci. Pollut. Res. Int..

[231] Wang X., Shen M., Zhou J., Jin Y. (2019). Chlorpyrifos Disturbs Hepatic Metabolism Associated with Oxidative Stress and Gut Microbiota Dysbiosis in Adult Zebrafish. Comp. Biochem. Physiol. Part-C Toxicol. Pharmacol..

[232] Condette C.J., Khorsi-Cauet H., Morlière P., Zabijak L., Reygner J., Bach V., Gay-Quéheillard J. (2014). Increased Gut Permeability and Bacterial Translocation After Chronic Chlorpyrifos Exposure in Rats. PLoS ONE.

[233] Tirelli V., Catone T., Turco L., Di Consiglio E., Testai E., De Angelis I. (2007). Effects of the Pesticide Clorpyrifos on an in vitro Model of Intestinal Barrier. Toxicol. Vitr..

[234] Liang Y., Zhan J., Liu D., Luo M., Han J., Liu X., Liu C., Cheng Z., Zhou Z., Wang P. (2019). Organophosphorus Pesticide Chlorpyrifos Intake Promotes Obesity and Insulin Resistance through Impacting Gut and Gut Microbiota. Microbiome.

[235] Fu H., Ge Y., Liu X., Deng S., Li J., Tan P., Yang Y., Wu Z. (2024). Exposure to the Environmental Pollutant Chlorpyrifos Induces Hepatic Toxicity through Activation of the JAK/STAT and MAPK Pathways. Sci. Total Environ..

[236] Han C., Sheng J., Pei H., Sheng Y., Wang J., Zhou X., Li W., Cao C., Yang Y. (2023). Environmental Toxin Chlorpyrifos Induces Liver Injury by Activating P53-Mediated Ferroptosis via GSDMD-MtROS. Ecotoxicol. Environ. Saf..

[237] Mackness M.I., Mackness B., Durrington P.N., Connelly P.W., Hegele R.A. (1996). Paraoxonase: Biochemistry, genetics and relationship to plasma lipoproteins. Curr. Opin. Lipidol..

[238] Hasselwander O., McMaster D., Fogarty D.G., Maxwell A.P., Nicholls D.P., Young I.S. (1998). Serum Paraoxonase and Platelet-Activating Factor Acetylhydrolase in Chronic Renal Failure. Clin. Chem..

[239] El-Banna S.G., Attia A.M., Hafez A.M., El-Kholy S.M. (2009). Effect of garlic consumption on blood lipid and oxidant/antioxidant parameters in rat males exposed to chlorpyrifos. Slovak J. Anim. Sci..

[240] Cheng W.W., Zhu Q., Zhang H.Y. (2019). Mineral Nutrition and the Risk of Chronic Diseases: A Mendelian Randomization Study. Nutrients.

[241] Metta V., Sanchez T.C., Padmakumar C. (2017). Osteoporosis: A Hidden Nonmotor Face of Parkinson’s Disease. International Review of Neurobiology.

[242] Watts N.B. (2014). Insights from the Global Longitudinal Study of Osteoporosis in Women (GLOW). Nat. Rev. Endocrinol..

[243] Ali S.J., Ellur G., Patel K., Sharan K. (2019). Chlorpyrifos Exposure Induces Parkinsonian Symptoms and Associated Bone Loss in Adult Swiss Albino Mice. Neurotox. Res..

[244] Sherif M.A., Carter W.G., Mellor I.R. (2025). Chlorpyrifos Acts as a Positive Modulator and an Agonist of N-Methyl-d-Aspartate (NMDA) Receptors: A Novel Mechanism of Chlorpyrifos-Induced Neurotoxicity. J. Xenobiot..

[245] Bright J.E., Inns R.H., Tuckwell N.J., Griffiths G.D., Marrs T.C. (1991). A histochemical study of changes observed in the mouse diaphragm after organophosphate poisoning. Hum. Exp. Toxicol..

[246] John M., Oommen A., Zachariah A. (2003). Muscle injury in organophosphorous poisoning and its role in the development of intermediate syndrome. Neurotoxicology.

[247] Kelly S.S., Mutch E., Williams F.M., Blain P.G. (1994). Electrophysiological and biochemical effects following single doses of organophosphates in the mouse. Arch. Toxicol..

[248] El Sabbouri H.E.K., Hallal N., Darwiche W., Gay-Quéheillard J., Bach V., Ramadan W., Joumaa W.H. (2022). Effect of Chronic Chlorpyrifos Exposure on Diaphragmatic Muscle Contractility and MHC Isoforms in Adult Rats. Toxicol. Environ. Health Sci..

[249] Darwiche W., Gay-Quéheillard J., Delanaud S., Sabbouri H.E.K.E., Khachfe H., Joumaa W., Bach V., Ramadan W. (2018). Impact of Chronic Exposure to the Pesticide Chlorpyrifos on Respiratory Parameters and Sleep Apnea in Juvenile and Adult Rats. PLoS ONE.

[250] Hallal N., El Sabbouri H.E.K., Salami A., Ramadan W., Khachfe H., Moustafa M.E., Khalil M., Joumaa W.H. (2019). Impacts of Prolonged Chlorpyrifos Exposure on Locomotion and Slow-and Fast- Twitch Skeletal Muscles Contractility in Rats. Toxicol. Rep..

[251] Chandra Sekaran S.P., Thotakura B., Jyothi A.K., Manickam S., Chanemougavally J., Prabhu K., Gopalan D.H. (2023). Effect of Chlorpyrifos and Its Metabolites on Skeletal System Development of Chick Embryo. Birth Defects Res..

[252] Balakrishnan P., Thirunavukarasu K., Tamizhmani P., Michael A.A., Velusamy T. (2025). Toxicological Impact of Chronic Chlorpyrifos Exposure: DNA Damage and Epigenetic Alterations Induces Neoplastic Transformation of Liver Cells. Biochem. Biophys. Res. Commun..

[253] Goldar S., Gachumi G., Siciliano S.D., Hogan N.S. (2024). The Role of Efflux Transporters in Cytotoxicity and Intracellular Concentration of Chlorpyrifos and Chlorpyrifos Oxon in Human Cell Lines. Toxicol. Vitr..

[254] Ventura C., Núñez M., Miret N., Martinel Lamas D., Randi A., Venturino A., Rivera E., Cocca C. (2012). Differential Mechanisms of Action Are Involved in Chlorpyrifos Effects in Estrogen-Dependent or -Independent Breast Cancer Cells Exposed to Low or High Concentrations of the Pesticide. Toxicol. Lett..

[255] Ventura C., Zappia C.D., Lasagna M., Pavicic W., Richard S., Bolzan A.D., Monczor F., Núñez M., Cocca C. (2019). Effects of the Pesticide Chlorpyrifos on Breast Cancer Disease. Implication of Epigenetic Mechanisms. J. Steroid Biochem. Mol. Biol..

[256] Modepalli N., Venugopal S.B. (2016). Clinicopathological Study of Surface Epithelial Tumours of the Ovary: An Institutional Study. J. Clin. Diagn. Res..

[257] Nishi K., Hundal S.S. (2013). Chlorpyrifos Induced Toxicity in Reproductive Organs of Female Wistar Rats. Food Chem. Toxicol..

[258] Wei W., Dizon D., Vathipadiekal V., Birrer M.J. (2013). Ovarian Cancer: Genomic Analysis. Ann. Oncol..

[259] Buján S., Pontillo C., Miret N., Leguizamón M.A., Chiappini F., Cocca C., Randi A. (2024). Triple Negative Breast Cancer Cells Exposed to Aryl Hydrocarbon Receptor Ligands Hexachlorobenzene and Chlorpyrifos Activate Endothelial Cells. Chem. Biol. Interact..

[260] Zárate L.V., Pontillo C.A., Español A., Miret N.V., Chiappini F., Cocca C., Álvarez L., de Pisarev D.K., Sales M.E., Randi A.S. (2020). Angiogenesis Signaling in Breast Cancer Models Is Induced by Hexachlorobenzene and Chlorpyrifos, Pesticide Ligands of the Aryl Hydrocarbon Receptor. Toxicol. Appl. Pharmacol..

[261] Moyano P., García J.M., García J., Pelayo A., Muñoz-Calero P., Frejo M.T., Anadon M.J., Naval M.V., Flores A., Mirat V.A. (2021). Chlorpyrifos Induces Cell Proliferation in MCF-7 and MDA-MB-231 Cells, Through Cholinergic and Wnt/β-Catenin Signaling Disruption, AChE-R Upregulation and Oxidative Stress Generation After Single and Repeated Treatment. Food Chem. Toxicol..

[262] Rich J.D., Gabriel S.M., Schultz-Norton J.R. (2012). In Vitro Effects of Herbicides and Insecticides on Human Breast Cells. ISRN Toxicol..

[263] Farhadi K., Tahmasebi R., Biparva P., Maleki R. (2015). In Vitro Study of the Binding Between Chlorpyrfos and Sex Hormones Using Headspace Solid-Phase Microextraction Combined with High-Performance Liquid Chromatography. Hum. Exp. Toxicol..

[264] Moyano P., García J., García J.M., Pelayo A., Muñoz-Calero P., Frejo M.T., Anadon M.J., Lobo M., Del Pino J. (2020). Chlorpyrifos-Induced Cell Proliferation in Human Breast Cancer Cell Lines Differentially Mediated by Estrogen and Aryl Hydrocarbon Receptors and KIAA1363 Enzyme After 24 h and 14 Days Exposure. Chemosphere.

[265] Ventura C., Venturino A., Miret N., Randi A., Rivera E., Núñez M., Cocca C. (2015). Chlorpyrifos Inhibits Cell Proliferation through ERK1/2 Phosphorylation in Breast Cancer Cell Lines. Chemosphere.

[266] Lerro C.C., Koutros S., Andreotti G., Friesen M.C., Alavanja M.C., Blair A., Hoppin J.A., Sandler D.P., Lubin J.H., Ma X. (2015). Organophosphate Insecticide Use and Cancer Incidence Among Spouses of Pesticide Applicators in the Agricultural Health Study. Occup. Environ. Med..

[267] Wang H., Ye Y., Zhu Z., Mo L., Lin C., Wang Q., Wang H., Gong X., He X., Lu G. (2016). MiR-124 Regulates Apoptosis and Autophagy Process in MPTP Model of Parkinson’s Disease by Targeting to Bim. Brain Pathol..

[268] Deddens J., Hines C. (2002). Biological and Air Monitoring of Chlorpyrifos Exposures Among Termiticide Applicators: Application of Mixed-Effect Models to Evaluate Exposure Determinants.

[269] Silva M.H. (2020). Effects of Low-Dose Chlorpyrifos on Neurobehavior and Potential Mechanisms: A Review of Studies in Rodents, Zebrafish, and Caenorhabditis Elegans. Birth Defects Res..

[270] Goodman J.E., Prueitt R.L., Rhomberg L.R. (2012). Incorporating Low-Dose Epidemiology Data in a Chlorpyrifos Risk Assessment. Dose-Response.

[271] Burns C.J., Garabrant D., Albers J.W., Berent S., Giordani B., Haidar S., Garrison R., Richardson R.J. (2006). Chlorpyrifos Exposure and Biological Monitoring Among Manufacturing Workers. Occup. Environ. Med..

[272] Agency for Toxic Substances and Disease Registry (US) (1997). Toxicological Profile for Chlorpyrifos.

[273] Barr D.B., Allen R., Olsson A.O., Bravo R., Caltabiano L.M., Montesano A., Nguyen J., Udunka S., Walden D., Walker R.D. (2005). Concentrations of Selective Metabolites of Organophosphorus Pesticides in the United States Population. Environ. Res..

[274] Fama F., Feltracco M., Moro G., Barbaro E., Bassanello M., Gambaro A., Zanardi C. (2023). Pesticides monitoring in biological fluids: Mapping the gaps in analytical strategies. Talanta.

[275] Andersen H.R., Rambaud L., Riou M., Buekers J., Remy S., Berman T., Govarts E. (2022). Exposure Levels of Pyrethroids, Chlorpyrifos and Glyphosate in EU—An Overview of Human Biomonitoring Studies Published since 2000. Toxics.

[276] Ayilara M.S., Adeleke B.S., Akinola S.A., Fayose C.A., Adeyemi U.T., Gbadegesin L.A., Omole R.K., Johnson R.M., Uthman Q.O., Babalola O.O. (2023). Biopesticides as a promising alternative to synthetic pesticides: A case for microbial pesticides, phytopesticides, and nanobiopesticides. Front. Microbiol..

[277] Fergani Y.A., Refaei E.A.E., Faiz N.M., Hamama H.M. (2023). Evaluation of Chlorpyrifos and *Beauveria bassiana* as a Strategy in the Egyptian Sugar Beet Fields: Impact on *Spodoptera littoralis* (Boisduval) and Its Associated Predators Populations and the Sugar Beetroot Yield. Egypt. J. Biol. Pest Control.

[278] Qureshi J.A., Kostyk B.C., Stansly P.A. (2014). Insecticidal suppression of Asian citrus psyllid *Diaphorina citri* (Hemiptera: Liviidae) vector of huanglongbing pathogens. PLoS ONE.

[279] Singh S., Gupta R., Sharma S. (2015). Effects of Chemical and Biological Pesticides on Plant Growth Parameters and Rhizospheric Bacterial Community Structure in Vigna Radiata. J. Hazard. Mater..

[280] Malinga L.N., Laing M.D. (2025). Efficacy of *Bacillus thuringiensis* and *Beauveria bassiana* in Controlling *Helicoverpa armigera*. Entomol. Appl. Sci. Lett..

[281] Acharya R., Sharma S.R., Barman A.K., Kim S.M., Lee K.Y. (2023). Control efficacy of azadirachtin on the fall armyworm, *Spodoptera frugiperda* (J. E. Smith) by soil drenching. Arch. Insect Biochem. Physiol..

[282] Liu K., Rao C., Li C., Jiang W., Hu B., Su J. (2025). Characterization of azadirachtin resistance in a laboratory-selected strain of *Drosophila melanogaster*. Pestic. Biochem. Physiol..

[283] Grimalt S., Thompson D.G., Coppens M., Chartrand D.T., Shorney T., Meating J., Scarr T. (2011). Analytical Study of Azadirachtin and 3-Tigloylazadirachtol Residues in Foliage and Phloem of Hardwood Tree Species by Liquid Chromatography—Electrospray Mass Spectrometry. J. Agric. Food Chem..

[284] European Food Safety Authority (EFSA) (2019). Statement on the available outcomes of the human health assessment in the context of the pesticides peer review of the active substance chlorpyrifos. EFSA J..

[285] European Commission (2020). Commission Implementing Regulation (EU) 2020/18 of 10 January 2020 concerning the non-renewal of the approval of the active substance chlorpyrifos-methyl. Off. J. Eur. Union.

[286] World Health Organization (WHO), Food and Agriculture Organization of the United Nations (FAO) (2023). Guidance on Use of Pesticide Regulation to Prevent Suicide.

[287] Health Canada (2021). Cancellation of Remaining Chlorpyrifos Registrations Under Paragraph 20(1)(a) of the Pest Control Products Act.

[288] US EPA (2021). Chlorpyrifos; Tolerance Revocations. Fed. Regist..

[289] PAN International (2024). Consolidated List of Banned Pesticides.

